# Globular Adiponectin Inhibits Breast Cancer Cell Growth through Modulation of Inflammasome Activation: Critical Role of Sestrin2 and AMPK Signaling

**DOI:** 10.3390/cancers12030613

**Published:** 2020-03-06

**Authors:** Duc-Vinh Pham, Pawan Kumar Raut, Mahesh Pandit, Jae-Hoon Chang, Nikita Katila, Dong-Young Choi, Jee-Heon Jeong, Pil-Hoon Park

**Affiliations:** College of Pharmacy, Yeungnam University, Gyeongsan 38541, Korea; vinhpd@hup.edu.vn (D.-V.P.); pawanraut73@gmail.com (P.K.R.); maheshpandit010@gmail.com (M.P.); jchang@yu.ac.kr (J.-H.C.); dychoi@yu.ac.kr (D.-Y.C.); nikitakatila28@gmail.com (N.K.); jeeheon@me.com (J.-H.J.)

**Keywords:** Adiponectin, Breast cancer, Inflammasomes, Sestrin2

## Abstract

Adiponectin, an adipokine predominantly derived from adipose tissue, exhibits potent antitumor properties in breast cancer cells. However, its mechanisms of action remain elusive. Inflammasomes—intracellular multimeric protein complexes—modulate cancer cell growth in a complicated manner, as well as playing a role in the innate immune system. Herein, we examined the potential role of inflammasomes in the antitumor activity of adiponectin and found that globular adiponectin (gAcrp) significantly suppressed inflammasomes activation in breast cancer cells both in vitro and in vivo conditions, as determined by decreased expression of inflammasomes components, including NOD-like receptor pyrin domain-containing protein 3 (NLRP3) and the apoptosis-associated speck-like protein containing a CARD (ASC), and inhibition of interleukin-1β and caspase-1 activation. Treatment with pharmacological inhibitors of inflammasomes caused decrease in cell viability, apoptosis induction, and G0/G1 cell cycle arrest, suggesting that inflammasomes activation is implicated in the growth of breast cancer cells. In addition, treatment with gAcrp generated essentially similar results to those of inflammasomes inhibitors, further indicating that suppression of breast cancer cell growth by gAcrp is mediated via modulation of inflammasomes. Mechanistically, gAcrp suppressed inflammasomes activation through sestrin2 (SESN2) induction, liver kinase B1 (LKB-1)-dependent AMP-activated protein kinase (AMPK) phosphorylation, and alleviation of endoplasmic reticulum (ER) stress. Taken together, these results demonstrate that gAcrp inhibits growth of breast cancer cells by suppressing inflammasomes activation, at least in part, via SESN2 induction and AMPK activation-dependent mechanisms.

## 1. Introduction

Adipocytes, a major constituent of adipose tissue, have been recently recognized as an active player in a wide variety of obesity-related endocrine and metabolic changes involved in tumorigenesis and malignant progression of various cancer types [1]. Compelling evidence indicated that the functions of adipose tissue other than energy storage are mediated by secretion of diverse biologically active molecules, collectively called adipokines. Among diverse adipokines, adiponectin is the most abundant in the circulation, which accounts for approximately 0.01% of total plasma proteins [1,2]. In addition to the full-length form, globular adiponectin, which contains only highly conserved globular domain of adiponectin, can be generated via proteolytic cleavage. Although the circulatory concentration of globular adiponectin is relatively low, it possesses potent diverse physiological activities [3]. 

Apart from its well-characterized physiological roles in lipid metabolism and insulin sensitization [4,5,6], adiponectin exhibits potent antitumor activities [7]. Notably, low plasma level of adiponectin or reduced expression of adiponectin receptors has been closely linked to increased risk of certain types of cancers [8,9,10]. The results obtained from a number of studies showed the anticancer activities of adiponectin are mediated through multiple mechanisms, including induction of apoptosis, cell cycle arrest, and inhibition of migration/invasion of cancer cells [11,12,13]. Mechanistically, many different molecular targets involved in these effects of adiponectin have been unveiled, such as AMPK activation, blocking leptin, and c-Jun NH2-terminal kinase 2 (JNK2) signaling; phosphorylation of extracellular signal-regulated kinases (ERK), the mitogen-activated protein kinase (MAPK), and protein phosphatase 2 A (PP2A); dephosphorylation of Akt; and induction of cytotoxic autophagy [14,15]. However, given that adiponectin regulates a wide variety of intracellular signaling pathways, the exact molecular mechanism underlying the inhibitory effects of adiponectin on cancer cell growth have not been completely understood.

Inflammation, one of the major hallmarks of cancer, mediates cancer progression via the coordinated actions of cytokines, chemokines, and innate immune cells [16]. Inflammasomes, cytosolic multiprotein oligomers containing a member of the NOD-like receptor protein (NLRP) family, the apoptosis-associated speck-like protein containing a CARD (ASC), and pro-caspase-1, are responsible for producing active interleukin family cytokines in response to infection and cellular stress [17]. In addition to the crucial roles in inflammatory and immune responses, emerging evidence has indicated that inflammasomes components are markedly upregulated in various cancers, such as lung, melanoma, oral squamous cell carcinoma, and breast cancer cells [18,19,20,21,22]. In addition, activation of the inflammasome facilitates tumor progression [23,24], suggesting that inflammasomes act as mediators that bridge chronic inflammation and tumorigenesis. Interestingly, in contrast to this notion, it has been also well documented that the suppressive effects on tumor growth by various anticancer agents are mediated through inflammasomes activation, which initiates pyroptotic cell death in cancer cells [25,26,27]. These conflicting reports suggest that the outcome of inflammasomes activation in growth of cancer cells would be specific for cell type, tumor tissue, and the nature of the inducers or inhibitors. Globular adiponectin has recently been shown to inhibit LPS-primed inflammasomes activation via autophagy induction and AMPK signaling in macrophages [28,29]. However, its effect in cancer cells and its implication in the regulation of cancer cell growth have not been explored. 

Sestrin2 (SESN2) is a member of a highly conserved stress-inducible gene family [30]. In addition to its critical roles in the homeostasis of cellular metabolism, SESN2 plays an important role in modulating survival and proliferation of cancer cells [31,32]. The modulatory role of SESN2 in determining the fate of the cells is mediated through AMPK phosphorylation [32,33]. Intriguingly, both SESN2 and AMPK act as modulators of inflammasomes activation in various cell types [34,35,36]. AMPK regulates inflammasomes through multiple mechanisms, including autophagy stimulation, maintenance of mitochondrial homeostasis, SIRT1 induction, or attenuation of the unfolded protein response (UPR) and C/EBP Homologous Protein (CHOP) expression in ER stress pathway [36,37]. Although it has been widely shown that SESN2/AMPK axis is implicated in various metabolic processes and determination of cellular fate, it remains unclear whether AMPK activation is required for SESN2-mediated inflammasomes suppression. Moreover, the involvement of SESN2 and AMPK signaling in adiponectin modulation of the inflammasome has not been investigated.

In this study, to elucidate the molecular mechanisms underlying antitumor activities of adiponectin, we examined the effect of globular adiponectin on inflammasomes activation and its role in the suppression of breast cancer growth. Herein, we found that inflammasome activation critically contributes to the growth of breast cancer cells by modulating both apoptosis and cell cycle. We have also demonstrated for the first time that globular adiponectin prevents NLRP3 inflammasomes activation in breast and hepatic cancer cells. In addition, suppressive effect of globular adiponectin on breast cancer cell growth is mediated through modulation of inflammasomes. Furthermore, suppression of inflammasomes activation by globular adiponectin is mediated via SESN2 induction, AMPK signaling, and inhibition of ER stress.

## 2. Results

### 2.1. Globular Adiponectin Inhibits Inflammasomes Activation in Cancer Cells

**Figure 1:**
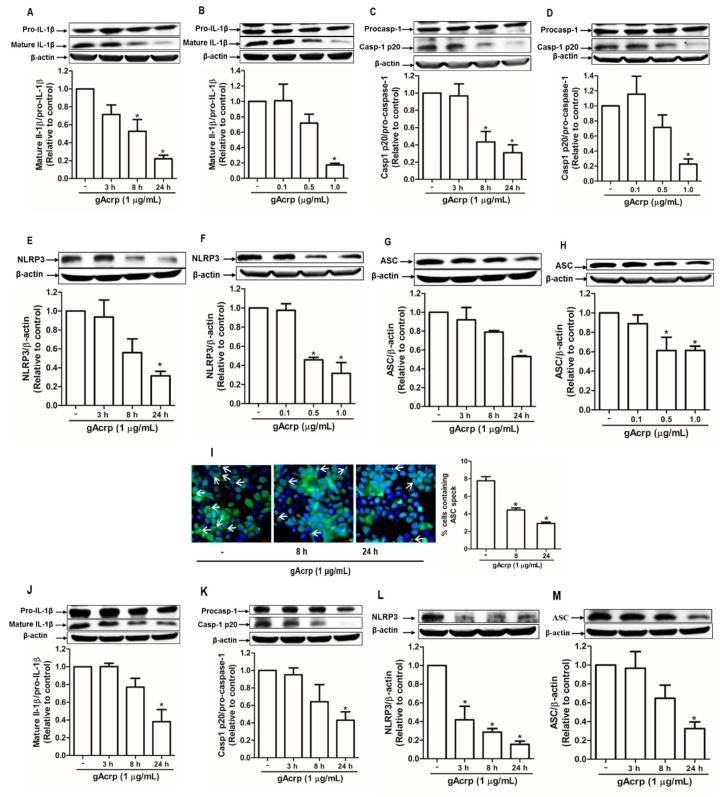
Suppression of the inflammasome activation by globular adiponectin in breast and hepatic cancer cells. (**A**–**H**) MCF-7 breast cancer cells were treated with gAcrp (1 µg/mL) for the indicated time duration (**A**,**C**,**E**,**G**) or treated with gAcrp at different concentrations for 24 h (**B**,**D**,**F**,**H**). Total protein lysates were immunoblotted for IL-1β (**A**,**B**), Caspase-1 (**C**,**D**), NOD-like receptor pyrin domain-containing protein 3 (NLRP3) (**E**,**F**), or the apoptosis-associated speck-like protein containing a CARD (ASC) (**G**,**H**). (**I**) MCF-7 cells were treated with gAcrp (1 µg/mL) for the indicated time periods before sequentially incubated with antibodies for ASC (green) and 4′,6-diamidino-2-phenylindole (DAPI) (blue). ASC speck formation was indicated by white arrows. Representative images from three independent experiments were presented along with the quantitation of ASC speck in the right panel. Values are expressed as percentage of the cells presenting the ASC dots with respect to DAPI by using Image Inside Software version 2.32. Scale bar: 5 µm. (**J**–**M**) HepG2 hepatic cancer cells were incubated with gAcrp (1 µg/mL) for time durations as indicated. Western blot analyses were performed for the measurement of IL-1β (J), caspase-1 (K), NLRP3 (L), and ASC (M). For Western blot experiments, representative images from three independent experiments are presented. β-actin was used as a loading control. Bar diagrams show the relative band intensity of the target proteins compared to β-actin or the respective proform, determined by densitometric analysis. Values are presented as the fold change compared with the control cells and are expressed as mean ± standard error of mean (SEM). * denotes *p* < 0.05 compared with control cells.

Inflammasomes activation requires a series of concerted actions leading to the maturation of interleukin family of proinflammatory cytokines, including interleukin-1β (IL-1β), as a final consequence. Therefore, to investigate the effect of gAcrp on cancer cell inflammasomes, we first examined the effect of gAcrp on the maturation of IL-1β and observed that gAcrp substantially decreased the level of active IL-1β in MCF-7 breast cancer cells in a time- and dose-dependent manner without significant effect on pro-IL-1β expression (Figure 1A,B). In addition, active caspase-1 (p20), which is responsible for the conversion of pro-IL-1β to mature IL-1β, was significantly suppressed by gAcrp treatment (Figure 1C,D). Globular adiponectin also markedly downregulated inflammasomes components, including NLRP3 and ASC, in MCF-7 cells (Figure 1E–H). During inflammasome activation, ASC is polymerized to form large helical fibrils called ASC specks, which function as adaptor molecules for recruitment of pro-caspase-1. As shown in Figure 1I, gAcrp treatment prominently decreased ASC speck formation in MCF-7 cells, further confirming the suppressive effect of gAcrp on inflammasomes activation. To further validate the effect of gAcrp on inflammasomes in different cancer cell types, we next examined the inflammasome activity under treatment with gAcrp in HepG2 hepatocellular carcinoma cells. In a similar way, gAcrp showed to inhibit the IL-1β maturation (Figure 1I), caspase-1 activation (Figure 1J), and the expression levels of NLRP3 (Figure 1K) and ASC (Figure 1L) in HepG2 cells. Taken together, these findings indicate that gAcrp suppresses activation of the NLRP3 inflammasome in cancer cells.

### 2.2. Modulation of Endoplasmic Reticulum Stress Is Implicated in the Suppression of the Inflammasome Activation by Globular Adiponectin in Breast Cancer Cells

**Figure 2:**
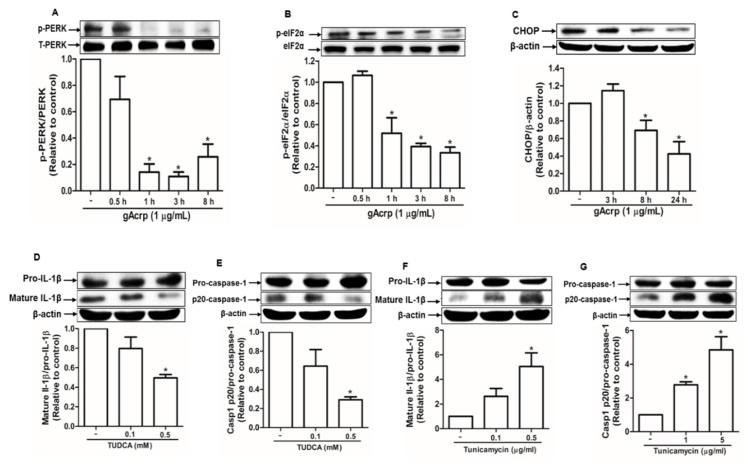
Suppression of ER stress by globular adiponectin and its implication in the modulation of inflammasomes activation in breast cancer cells. (**A**–**C**) MCF-7 cells were treated with gAcrp (1 µg/mL) for the indicated time duration. Expression levels of phospho- and total protein kinase RNA-like endoplasmic reticulum kinase (PERK) (**A**), phospho- and total eukaryotic translation initiation factor 2A (eIF2α) (B), and C/EBP homologous protein (CHOP) were determined by Western blot analysis. (**D**–**G**) MCF-7 cells were incubated with the indicated concentrations of tauroursodeoxycholic acid (TUDCA) (**D**,**E**) or tunicamycin (**F**,**G**) for 24 h or 12 h, respectively. Immunoblot analysis was carried out for determining the levels of interleukin-1β (IL-1β) and caspase-1. For all the Western blot analyses, the expression level of the target genes was estimated by densitometric analysis and is shown in the lower panel. Values represent fold change in comparison to the control group after being normalized to β-actin and are expressed as mean ± standard error of mean (SEM), *n* = 3. * denotes *p* < 0.05 compared with control cells.

ER stress, which is usually upregulated in cancer cells, contributes to inflammasome activation [38]. To investigate the mechanisms underlying inhibition of inflammasome activation by gAcrp, we assessed the effect of gAcrp on ER stress and its potential role in the modulation of inflammasomes activation. As shown in Figure 2, gAcrp inhibited the protein kinase RNA-like endoplasmic reticulum kinase (PERK) arm of the unfolded protein response in ER stress signaling cascade in MCF-7 cells. In particular, gAcrp treatment significantly reduced the phosphorylation of PERK (Figure 2A) and its downstream kinase, eIF2α (Figure 2B), in a time-dependent manner. Moreover, the expression level of CHOP was decreased by treatment with gAcrp (Figure 2C). To further understand the role of ER stress regulation in gAcrp-inhibition of inflammasome activation, we evaluated the effects of ER stress modulators on IL-1β maturation and caspase-1 activation in MCF-7 cells. Tauroursodeoxycholic acid (TUDCA), a classical inhibitor of ER stress, significantly reduced the levels of mature IL-1β (Figure 2D) and active subunit of caspase-1 (p20) (Figure 2E) in a dose-dependent manner. On the contrary, tunicamycin, a pharmacological ER stress inducer, induced significant increases in mature IL-1β and active caspase-1 in MCF-7 cells (Figure 2F,G). Collectively, these results suggest that ER stress contributes to inflammasomes activation and that alleviation of ER stress would be a potential mechanism for suppression of inflammasomes activation by gAcrp in breast cancer cells.

### 2.3. AMPK Plays an Integral Role in the Modulation of Inflammasomes Activation and ER Stress by Globular Adiponectin in Breast Cancer Cells

**Figure 3:**
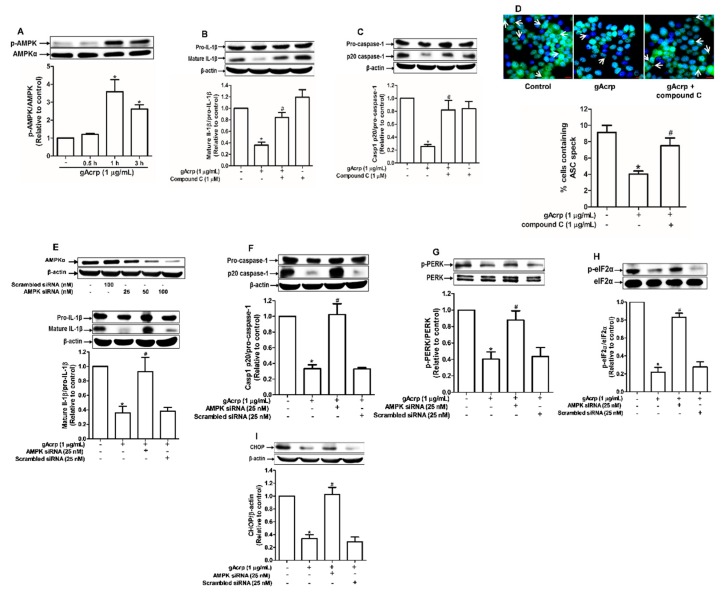
Crucial role of AMP-activated protein kinase (AMPK) signaling in the modulation of endoplasmic reticulum (ER) stress and inflammasomes by globular adiponectin. (**A**) MCF-7 cells were treated with gAcrp (1 µg/mL) for the indicated time periods. The expression levels of AMPKα were measured by Western blot analysis. (**B**,**C**) MCF-7 cells were pretreated with compound C (1 µM), a pharmacological inhibitor of AMPK, for 1h followed by further treatment with gAcrp (1 µg/mL) for additional 24 h. Total protein lysates were prepared and used for immunoblotting analysis for the measurement of interleukin-1β (IL-1β) (**B**) and Caspase-1 (**C**). (**D**) After pretreatment with compound C for 1 h, MCF-7 cells were incubated with gAcrp (1 µg/mL) for additional 1 h and further incubated with antibodies for specific ASC (green) and DAPI (blue). Scale bar: 5µm. (**E**–**I**) MCF-7 cells were transfected with siRNA targeting AMPKα or control scrambled siRNA. Gene silencing efficiency of AMPKαwas monitored by Western blot analysis (Upper panel in Figure 3E). After transient gene silencing of AMPKα, cells were treated with gAcrp for 24h (**E**,**F**) or 1 h (**G**–**I**). IL-1β (E), Caspase-1 (**F**), protein kinase RNA-like endoplasmic reticulum kinase (PERK) (G), Eukaryotic translation initiation factor 2A (eIF2α) (**H**), and C/EBP homologous protein (CHOP) (**I**) expression levels were determined by Western blot analysis. Values are presented as the fold change compared with the control cells and are expressed as mean± standard error of mean (SEM), *n*= 3. * denotes *p* < 0.05 compared to control cells, # denotes *p* < 0.05 compared with the cells treated with globular adiponectin but not pretreated with compound C (**B**,**C**) or not transfected with siRNA (**E**–**I**).

AMPK acts as a master sensor of various biological responses induced by adiponectin. To further clarify the mechanisms involved in inflammasomes inhibition, we examined whether AMPK mediates the inhibitory effects of gAcrp on inflammasomes and ER stress. Treatment with gAcrp induced phosphorylation of AMPK in MCF-7 cells (Figure 3A), consistent with previous reports. Notably, inhibition of AMPK signaling by either a pharmacological inhibitor (compound C) (Figure 3B,C) or gene silencing of AMPKα (Figure 3E,F) led to restoration of mature IL-1β and caspase-1 levels in gAcrp-treated MCF-7 cells. Compound C also abrogated the inhibitory effect of gAcrp on ASC speck formation (Figure 3D). Given that relief of ER stress is involved in the regulation of inflammasomes by gAcrp, we further investigated contribution of AMPK signaling in inhibition of ER stress. As expected, transfection with a siRNA targeting AMPKα restored the phosphorylation of PERK (Figure 3G) and eIF2α (Figure 3H), and the expression level of CHOP (Figure 3I) in MCF-7 cells treated with gAcrp. Taken together, these findings support the notion that AMPK signaling plays a key role in the regulation of ER stress and inflammasomes by gAcrp.

### 2.4. Sestrin2 Acts as an Upstream Signaling Molecule for AMPK Activation and Mediates the Modulatory Effects of Globular Adiponectin on ER Stress and Inflammasomes

**Figure 4:**
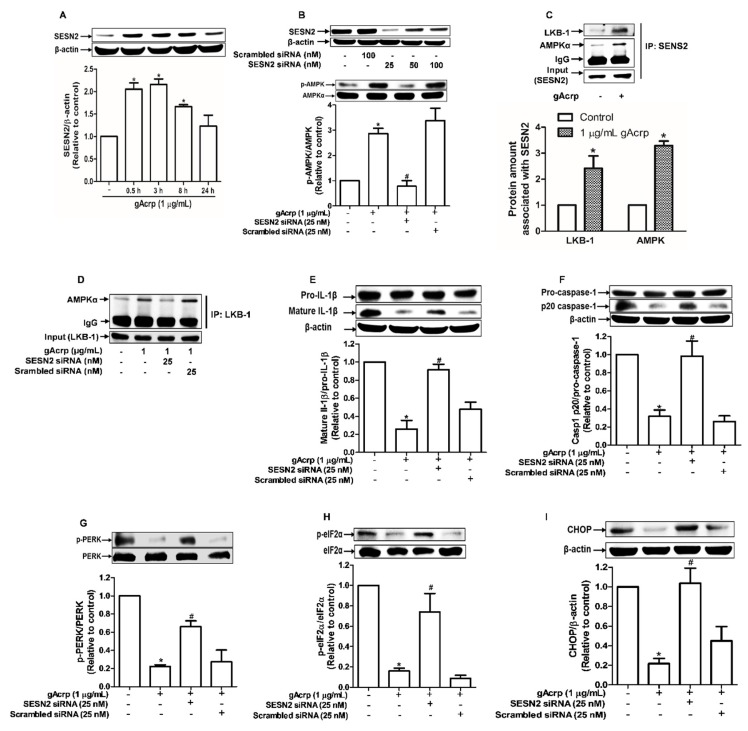
Role of sestrin2 (SESN2) induction in AMP-activated protein kinase (AMPK) phosphorylation, endoplasmic reticulum (ER) stress amelioration, and inflammasome inhibition by globular adiponectin in breast cancer cells. (**A**) MCF-7 cells were treated with gAcrp (1 µg/mL) for the indicated time periods. The expression level of SESN2 was determined by Western blot analysis. (**B**) MCF-7 cells were transiently transfected with siRNA targeting AMPKα and the gene silencing efficiency was monitored after 36 h using immunoblotting analysis (upper panel). After transfection, MCF-7 cells were further incubated with gAcrp (1 µg/mL) for 1 h and the expression of AMPK was examined (lower panel). (**C**) MCF-7 cells were treated with gAcrp (1 µg/mL) for 30 min. The SESN2-associated protein levels of AMPK and LKB-1 were determined by Immunoprecipitation assay. (**D**) After transfection with SESN2 siRNA, MCF-7 cells were further incubated with gAcrp (1 µg/mL) for 30 min. AMPKα amount associated with liver kinase B1 (LKB-1) was measured by immunoprecipitation assay. (**E**–**I**) MCF-7 cells were transfected with siRNA targeting SESN2 or control scrambled siRNA followed by treatment with gAcrp (1 µg/mL) for 1 h (G,H) or 24 h (**E**,**F**,**I**). Total protein lysates were immunoblotted for interleukin-1β (IL-1β) (E), Caspase-1 (**F**), the protein kinase RNA-like endoplasmic reticulum kinase (PERK) (**G**), Eukaryotic translation initiation factor 2A (eIF2α) (**H**), and C/EBP Homologous Protein (CHOP) (**I**). Values represent the fold change relative to the control cells and are expressed as mean± standard error of mean, *n* = 3. * denotes *p* < 0.05 compared to control cells, # denotes *p* < 0.05 compared with the cells treated with gAcrp but not transfected with siRNA.

Sestrin2 (SESN2), a member of stress-inducible metabolic proteins, is considered one of the critical regulators of ER stress and AMPK activation [33,39]. To further clarify the upstream signaling mechanisms by which gAcrp suppresses inflammasomes activation in cancer cells, we examined the role of SESN2 signaling. As shown in Figure 4A, gAcrp treatment induced a marked increase in SESN2 protein expression, whereas the mRNA expression level was not significantly affected (Figure S1), indicating that gAcrp upregulates SESN2 expression through a post-transcriptional mechanism. SESN2 knockdown led to prevention of gAcrp-stimulated AMPK phosphorylation in MCF-7 cells (Figure 4B), implying that SESN2 mediates gAcrp-induced AMPK phosphorylation. As SESN2 would not directly phosphorylate AMPK, we assume that SESN2 induction leads to AMPK phosphorylation by an indirect manner. As shown in Figure 4C, gAcrp treatment significantly enhanced complex formation of SESN2/AMPK and SESN2/LKB-1, an upstream kinase of AMPK, as determined by immunoprecipitation using SESN2 antibody. Furthermore, although gAcrp induced a substantial increase in association of LKB1/AMPK, gene silencing of SESN2 almost completely abolished gAcrp-stimulated formation of this complex (Figure 4D). All these results suggest that SESN2 acts as a scaffold for AMPK and LKB-1, which causes subsequent phosphorylation of AMPK. In subsequent experiments verifying the functional role of SESN2 in the modulation of inflammasomes and ER stress by gAcrp, reduction in IL-1β maturation and caspase-1 activation by gAcrp were markedly recovered by gene silencing of SESN2 in MCF-7 cells (Figure 4E,F). Likewise, gene silencing of SESN2 prevented ER stress amelioration by gAcrp, as demonstrated by restoration of the PERK and eIF2α phosphorylation, and CHOP expression (Figure 4G–I). Collectively, these results reveal that SESN2 induction critically contributes to AMPK activation, ER stress homeostasis, and finally suppression of inflammasomes activation in breast cancer cells.

### 2.5. Suppression of Breast Cancer Cell Growth by Globular Adiponectin is Mediated through Modulation of Inflammasomes Activation

**Figure 5:**
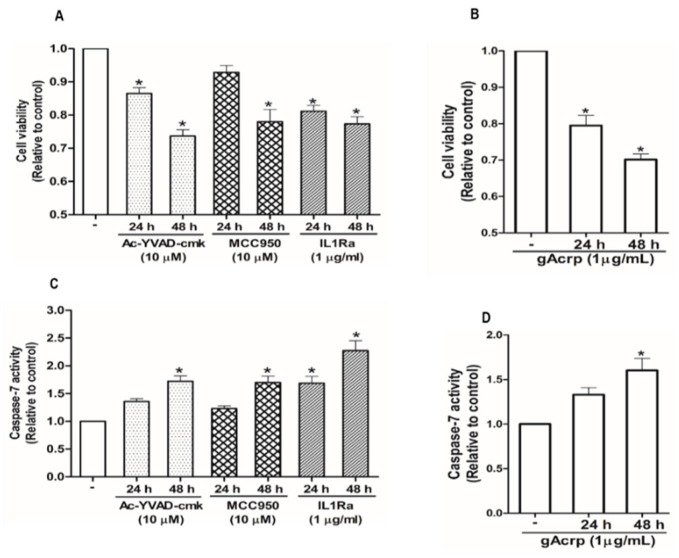

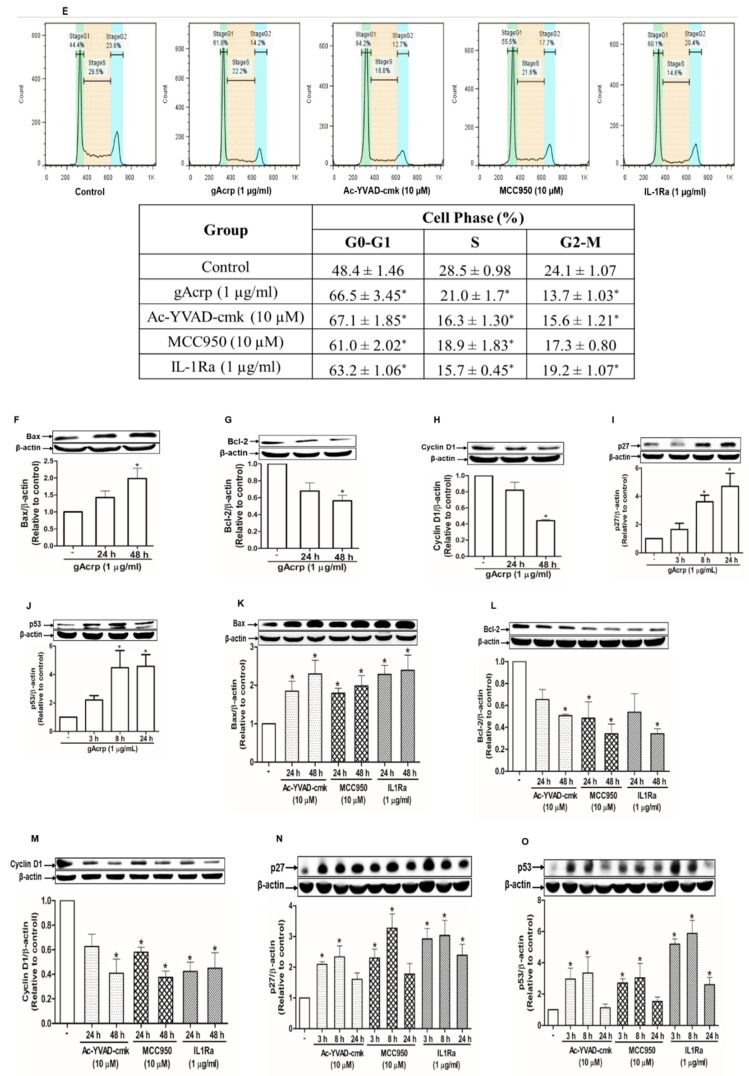
Inhibition of the inflammasome activation mediates modulation of breast cancer cell growth. (**A**–**D**) MCF-7 cells were treated with pharmacological inhibitors of inflammasomes (**A**,**C**), including Ac-YVAD-cmk (10 µM), a caspase-1 inhibitor, MCC950 (10 µM), a small molecule inhibitor of NLRP3, or interleukin-1 receptor antagonist (1 µg/mL), or gAcrp (1 µg/mL) (**B**,**D**) for the indicated time duration. Cell viability (**A**,**B**) and Caspase-7 enzyme activity (**C**,**D**) were then determined as described in the Materials and Methods. (**E**) MCF-7 cells were incubated with gAcrp (1 µg/mL) or pharmacological inhibitors of inflammasomes for 48 h followed by staining with propidium iodide and flow cytometric analysis. (**F**–**O**) MCF-7 cells were incubated with gAcrp (1 µg/mL) (**F**–**J**) or pharmacological inhibitors of inflammasomes (**L**–**O**) for the indicated time periods. Immunoblot analysis was performed to determine the expression levels of Bcl2-associated X protein (Bax) (**F**,**K**), B-cell lymphoma 2 (Bcl-2) (**G**,**L**), cyclin D1 (**H**,**M**), p27 (**I**,**N**), and p53 (**J**,**O**). Images are the representative for three independent experiments that showed similar results. Values represent the fold change relative to the control cells and are expressed as mean± standard error of mean, *n* =3. * denotes *p* < 0.05 compared to control cells.

Inflammasomes activation is implicated in the pathogenesis of a variety of tumors in a complicated manner. We speculated that there was a link between attenuation of inflammasomes activation and the suppression of breast cancer cell growth by gAcrp. For this, we first confirmed that various pharmacological inhibitors of inflammasomes, including Ac-YVAD-cmk and MCC950, selective inhibitors of caspase-1 and NLRP3, respectively, and interleukin-1 receptor antagonist (IL-1Ra) significantly decreased the cell viability of MCF-7 (Figure 5A) and T47D breast cancer cells (Figure S2A) but not triple-negative MDA-MB-231 breast cancer cells (Figure S2B), suggesting that inflammasomes activation drives growth of estrogen receptor (ER)-positive breast cancer cells in our experimental conditions. Globular adiponectin treatment generated results largely similar to the treatment with inflammasomes inhibitors (Figure 5B). To further clarify how inflammasomes activation induces breast cancer cell growth, we examined the effects of gAcrp and inflammasomes inhibitors on apoptosis rate and cell cycle progression. As depicted in Figure 5C,D, pharmacological inhibitors of inflammasomes and gAcrp increased the caspase-7 activity in MCF-7 cells. In addition, gAcrp and inflammasomes inhibitors were found to induce cell cycle arrest at the G0/G1 phase (Figure 5E). These results were further confirmed by measuring the expression levels of the genes related with cell proliferation and apoptosis. Accordingly, treatment with gAcrp or inflammasomes inhibitors were found to upregulate proapoptotic genes, Bax, and negative modulators of the cell cycle, including p27^Kip^ and p53, but downregulate Bcl-2, an antiapoptotic protein, and cyclin D1 (Figure 5F). Taken together, these findings clearly demonstrate that inflammasomes activation contributes to the growth of breast cancer cells by suppressing apoptosis and cell cycle progression, and further suggest that suppressive effects of gAcrp on breast cancer growth would be mediated by suppressing inflammasomes activation.

### 2.6. In Vivo Effects of Globular Adiponectin on NLRP3 Inflammasome Activation that Mediates Tumor Growth in an MCF-7 Tumor Xenograft Model

**Figure 6:**
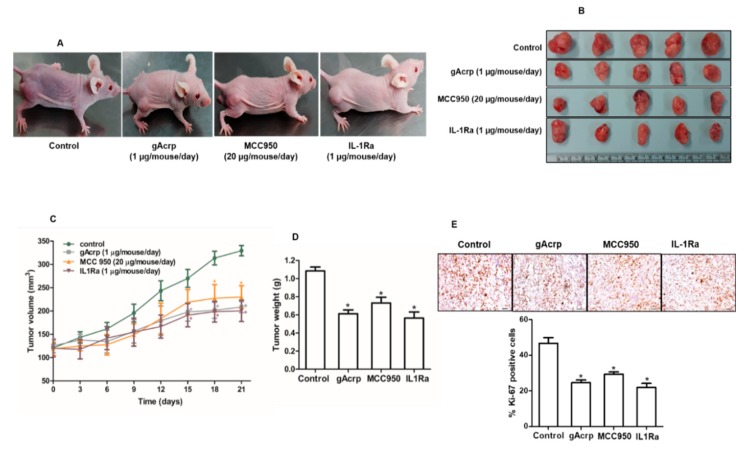

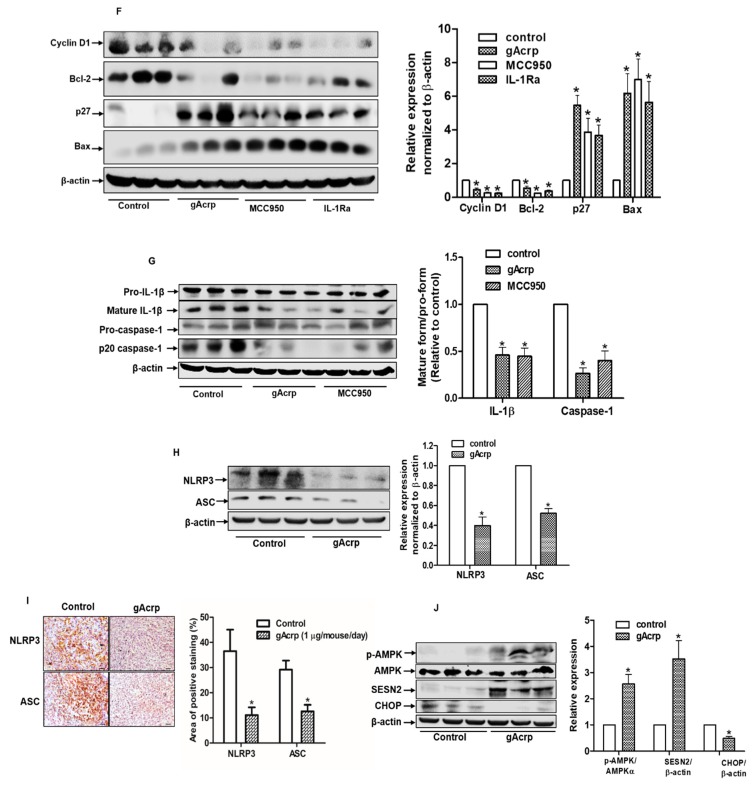
Suppressive effects of globular adiponectin on NLRP3 inflammasomes activation in MCF-7 tumor xenograft model and its potential role in the modulation of growth of breast cancer cells. After xenograft tumors were generated in BALB/c nude male mice, the animals were randomly divided into four groups of 5 mice and intratumorally administered with gAcrp (2µg/mouse), MCC950 (40µg/mouse), IL-1Ra (2µg/mouse) or phosphate-buffered saline (control group) every 48 h for 3 weeks. (**A**) Representative images of mice bearing xenografted tumors from each group at the end of the treatment period. (**B**) Tumor tissues were isolated from each mouse after 3 weeks of treatment. (**C**) Tumor growth rate were monitored every three days during treatment. (**D**) After 3 weeks treatment, tumor tissues from each mouse were collected and weighed. (**E**) Tissue sections were prepared from tumor of each mouse and subjected to immunohistochemistry (IHC) staining for Ki-67. Scale bar: 5µm. (**F**–**H**) The expression levels of the proliferative and apoptotic markers, such as cyclin D1, p27, B-cell lymphoma 2 (Bcl-2), and Bcl2-associated X protein (Bax) (Figure 6F), and genes related to inflammasomes, including IL-1β and caspase-1 (Figure 6G), NLRP3, and the apoptosis-associated speck-like protein containing a CARD (ASC) (Figure 6H), were determined by Western blot. (**I**) The expression levels of NLRP3 and ASC were further confirmed by IHC. For IHC experiments, five mice were used in each group and statistical analysis was made with 3 different tumor sections of each animal. The percentage of staining positive cells (nuclear staining) or staining positive area (cytoplasmic staining) was determined using Image J Software version 1.52. Magnification 20×. Scale bar: 5µm. (**J**) Expression levels of AMP-activated protein kinase (AMPK), sestrin2 (SESN2), and C/EBP homologous protein (CHOP) were examined by Western blot analysis. For Western blot analyses, 5 mice were used in each group and the results from each mouse were included in the statistical analysis. Of the five samples, representative images for three samples in each group were presented. Values are expressed as mean± standard error of mean, *n* = 5. * denotes *p* < 0.05 compared with control group.

The in vivo modulatory effect of gAcrp on inflammasomes activation and its critical role in the suppression of breast cancer cells growth by gAcrp were further validated in an MCF-7 tumor xenograft model established in BALB/c nude mice. As shown in Figure 6, treatment with gAcrp substantially inhibited tumor growth as evidenced by decreases in tumor size, tumor volume, and tumor weight (Figure 6B–D), consistent with our in vitro data and previous reports. Interestingly, essentially identical effects were observed upon intratumoral administration of an inhibitor of NLRP3 inflammasomes (MCC950) or an IL-1 receptor antagonist (IL1-Ra), indicating the critical role of inflammasomes activation and IL-1β signaling in tumor growth in an MCF-7 xenograft model. Subsequent analyses further revealed that administration of gAcrp and inflammasomes inhibitors suppressed cellular proliferative activity and induced apoptosis in the tumor tissue. In particular, gAcrp, MCC950, and IL-1Ra decreased expression of Ki-67 and cyclin D1, markers of cell proliferation, but increased expression of p27^Kip1^, an inhibitor of cell cycle progression (Figure 6E,F). In contrast, Bax expression was upregulated, while Bcl-2 expression was downregulated by treatment with gAcrp, MCC950, or IL-1Ra (Figure 6F). We finally verified whether gAcrp suppresses inflammasomes activation in tumor tissues and found that gAcrp reduced the expression levels of active IL-1β and p20 caspase-1 (Figure 6G). In addition, expression levels of inflammasomes components, including NLRP3 and ASC, were downregulated, as evidenced by Western blot (Figure 6H) and immunohistochemistry (Figure 6I) analyses. Furthermore, in line with our in vitro findings, treatment with gAcrp led to increased AMPK phosphorylation and SESN2 expression, but decreased CHOP expression (Figure 6J), which confirms the involvement of SESN2/AMPK/ER stress signaling pathway in the suppression of inflammasomes and tumor growth by adiponectin.

## 3. Discussion

Adiponectin, the most abundant adipokine, exhibits diverse physiological properties. In addition to insulin-sensitizing, lipid metabolism, and anti-inflammatory properties, recent studies have revealed that adiponectin possesses potent tumor growth-limiting effects through multiple mechanisms. Moreover, decrease in plasma adiponectin correlates to cancer development. Based on previous reports, although it is widely accepted that adiponectin inhibits tumor growth, its underlying mechanisms remain elusive. Chronic inflammation in tumor microenvironments has impacts on the development and progression in various types of tumors. Inflammasomes, composed of multiprotein subunits, are activated upon infection or cellular stress and act as a platform for the production of active IL-1 family inflammatory cytokines. Interestingly, recent study clearly indicates that inflammasomes are implicated in the modulation of cancer cell growth in a complicated manner, as well as acting as an essential component of the immune system. Inflammasomes activation in response to a wide variety of endogenous or exogenous stimuli facilitates a programmed cell death, called pyroptosis via induction of caspase-1 activation. Therefore, inflammasomes have been reported to prevent cancer development and activation of inflammasomes was attributed to a mechanism for the anticancer agents, such as induction of pyroptosis by omega-3 docosahexaenoic acid in triple-negative breast cancer cells [27] and inhibition of hepatocellular carcinoma cell growth by estradiol [40]. However, in contrast to this notion, there has been an increasing appreciation that excessive inflammasomes activation could contribute to growth of the multiple types of cancer cells [18,19,22]. These contradictory reports imply that role of the inflammasome in tumor growth would be depending on the experimental conditions, including types and stages of the tumors, and the downstream molecules involved. In the present study, we have examined if inflammasomes are implicated in modulation of tumor growth by adiponectin. Herein, we have demonstrated that inflammasomes activation crucially contributes to the growth of breast cancer cells by suppressing apoptosis and inducing cell cycle progression. Moreover, inhibition of breast cancer cells growth by globular adiponectin is mediated, at least in part, via suppression of inflammasomes activation.

Inflammasome activation is initiated with oligomerization of NOD-like receptor protein (NLR), followed by sequential recruitment of ASC and pro-caspase-1, finally leading to formation of cleaved caspase-1. Caspase-1 subsequently catalyzes the conversion of pro-IL-1β to mature IL-1β [41]. To investigate the role of inflammasome signaling in modulation of breast cancer cell growth, we used three inflammasome inhibitors, which act at different stages of inflammasome activation process. Although MCC950, a potent small molecule inhibitor of NLRP3 inflammasome, was previously reported to inhibit survival, migration, and invasion in head, neck, and lung cancer [42,43], the effect of caspase-1 inhibitors, such as Ac-YVAD-cmk, on tumor growth remains controversial. In a study performed by Sun et al., treatment with Ac-YVAD-cmk markedly increased cell proliferation and invasion, and decreased apoptotic levels in MDA-MB-231 breast cancer cells [44]. By contrast, the inhibition of caspase-1 by Ac-YVAD-CHO led to apoptosis in pancreatic carcinoma cells [45], implying that the role of caspase-1 in the survival and growth of cancer cells is also depending on the experimental condition, particularly determined by cancer cell types. According to our observations, treatment with inhibitor of NLRP3 inflammasome or caspase-1 (MCC950 or Ac-YVAD-cmk) showed to inhibit breast cancer cells growth in MCF-7 and T47D breast cancer cells. In addition, treatment with IL-1 RA resulted in the essentially similar responses, suggesting that inflammasomes activation and IL-1β secretion have a tumor promoting effect. Although numerous studies have highlighted the contributions of inflammasomes to carcinogenesis, little effort has been made to develop anticancer agents that modulate inflammasomes. In fact, to date, no anticancer agent directly targeting inflammasomes is available. Our present findings may open a new approach for potential therapeutic strategies for the treatment of breast cancer.

Notably, previous studies have indicated that caspase-1 activation and IL-1β secretion contribute to pyroptotic cell death in estrogen receptor (ER)-negative breast cancer cells [27,44], which are contrary to our present observations in ER-positive breast cancer cells. Indeed, the tumor growth suppressing effect of inflammasomes inhibitors was not detected in triple-negative MDA-MB-231 cells in our study (Figure S2B), suggesting a possibility that ERα signaling plays a role in the linkage between inflammasomes and breast tumor growth. This notion is supported by previous reports demonstrating that ERα signaling mediates the growth of breast cancer cells under various pathological conditions, including obesity [46,47] and crucially contributes to inflammasomes activation and tumor growth induced by leptin, another well-known adipokine [48,49]. Further studies will be needed to gain insights into the role of ER signaling in differential role of inflammasomes signaling in breast cancer growth. 

A large body of clinical epidemiological evidence has indicated the adiposity–cancer associations in which obesity has been considered as a significant risk factor for cancer initiation as well as a substantial driver of tumor progression [49,50]. Although various mechanisms, such as chronic inflammation, sex hormone deregulation, and adipokine pathophysiology, have been proposed for the linkage between adiposity and cancer [51,52], the underlying molecular mechanisms still remain poorly understood. Emerging evidence also reveals that adipose tissue-associated inflammation is a major mechanism by which obesity promotes metabolic disorders and cancer risk [53,54]. Therefore, the role of inflammasomes, a key player in inflammatory and immune responses, in regard to obesity-related diseases, has been being paid increasing attention. For instance, NLRP3 inflammasome is upregulated in differentiating adipocytes and elimination of NLRP3 inflammasome protects against obesity-induced pancreatic damage and metabolic dysfunction [55,56,57]. Likewise, increased expression of NLRC4 was found in tumor tissues from obese breast cancer patients and NLRC4 inflammasome is required for obesity-driven breast-cancer progression in diet-induced obese mice [22]. The importance of inflammasome signaling in the connection of obesity with cancer and the fact that adiponectin levels declined in obese patients strongly suggest potential involvement of inflammasome modulation in antitumor activities of adiponectin [58]. In this study, we demonstrate that inflammasomes suppression is a novel mechanism underlying the tumor growth-limiting effects of adiponectin. In addition to breast cancer cells, adiponectin was found to suppress inflammasomes activation in human hepatocarcinoma cancer cells (Figure 1), suggesting that inflammasomes modulation might be a common response by adiponectin in various cancer cells. To the best of our knowledge, this is the first report demonstrating the involvement of inflammasomes in the suppressive effect of adiponectin on tumor growth. It is also interesting to note that leptin, another well-known adipokine, promotes breast tumor growth through NLRP3 inflammasomes activation [49]. Given the previous reports that the plasma levels of adiponectin and leptin are differentially modulated in obese patients and these adipokines often show opposites activities on tumor development [59], the findings of this study suggest that differential regulation of inflammasomes activation by adiponectin and leptin could be a potential mechanism underlying the contrasting effects of these adipokines. Furthermore, as alterations in adipokine secretion are referred to elevated risk of various cancers in postmenopausal women with overweight and obesity [60], targeting inflammasomes might be a potential intervention for breaking the link between obesity and breast cancer mediated by adipokines.

In an attempt to clarify molecular mechanisms for the modulation of inflammasome activation by adiponectin, we found that SESN2 is essentially required for adiponectin suppression of inflammasomes activation. SESN2, a member of stress-inducible gene family, has been shown to play a role in the regulation of cell viability and proliferation under different cellular stressors. In many cancer cells, overexpression of SESN2 contributes to inhibition of cell growth through AMPK phosphorylation, the mammalian target of rapamycin (mTOR) inhibition, and autophagy induction [31,32]. A recent study reported that SESN2 suppressed sepsis by inhibiting NLRP3 activation and inducing autophagy in macrophages [34]. Note that the physiological roles of SESN2 appear to connect with that of adiponectin signaling. In this study, we have demonstrated that SESN2 induction plays a critical role in AMPK phosphorylation and amelioration of ER stress by gAcrp, and further elucidated an integral contribution of SESN2 signaling to suppression of the inflammasome activation by adiponectin. Although SESN2 is widely known as a potential therapeutic target for various types of cancer, to the best of our knowledge, this is first report to demonstrate the involvement of SESN2 signaling in the antitumor activity by adiponectin. Although, at this stage, we did not fully address the mechanisms underlying SESN2 induction by adiponectin, we found that gAcrp did not affect SESN2 mRNA levels (Figure S1), implying that gAcrp-induced SESN2 expression is mediated through post-transcriptional mechanism, which is consistent with previous reports showing that the increased SESN2 expression in LPS-stimulated macrophages is independent of transcriptional activity [34], and that SESN2 levels are modulated by phosphorylation and proteasomal degradation [61,62]. Given that SESN2 plays a pivotal role in the modulation of inflammasomes activation and tumor growth by gAcrp, elucidation of the molecular basis by which adiponectin induces SESN2 expression would provide a potential therapeutic target for the treatment of cancer. 

AMPK has been well recognized as an important integrator in a wide range of biological activities of adiponectin, including lipid metabolism, insulin sensitization, and control of growth and apoptosis in cancer cells. Previous studies reported that adiponectin-induced AMPK phosphorylation is mediated by LKB-1 [63]. Although LKB-1 has been shown to act as an upstream kinase for AMPK phosphorylation by gAcrp, the underlying mechanisms are not fully elucidated. Herein, we speculated if SESN2 mediates LKB1-induced AMPK phosphorylation in gAcrp-treated MCF-7 cells and found that gAcrp treatment prominently enhanced complex formation of LKB1/SESN2, AMPK/SESN2, and LKB1/SESN2 determined by immunoprecipitation assay. Interestingly, knockdown of SESN2 abrogated association of LKB1 and AMPK and prevented gAcrp-induced phosphorylation of AMPK (Figure 4C,G). In fact, in this study, adiponectin was found to increase the binding ability of SESN2 with LKB-1 and AMPK, as well as increasing the expression level. These results demonstrate that SESN2 is required for AMPK activation process, where it serves as a scaffold for interaction of LKB-1 with AMPK, and SESN2 mediates the effects of adiponectin on inflammasomes and cell survival/growth by facilitating phosphorylation of AMPK.

In the present study, we clearly demonstrated that AMPK activation is an essential event for the suppression of inflammasomes by adiponectin. Although previous studies have indicated that AMPK signaling is implicated in the modulation of the inflammasome in immune cells via upregulation of AMPK-autophagy axis and modulation of AMPK-reactive oxygen species (ROS) pathway [28,64], the role of AMPK signaling in modulation of inflammasomes activation in cancer cells has not been well defined. With regards to the relationship between AMPK and inflammasomes, recent studies have shown that AMPK activation may lead to inhibition of the NLRP3 inflammasome through various mechanisms. Herein, we examined the involvement of ER stress modulation in this process. There has been increasing evidence demonstrating that ER stress plays a role in controlling inflammasomes activation. In addition, ER stress marker genes are commonly overexpressed in tumor tissues and abnormally highly regulated ER stress leads to proliferation of the cancer cells [65,66]. AMPK signaling has been also recently shown to suppress NLRP3 inflammasome by regulating ER stress. In fact, a number of pharmacological agents possessing antitumor activity, such as β-hydroxybutyrate, metformin, resveratrol, salicylate, or AICAR, suppress inflammasomes activation by modulating AMPK signaling and ER stress [38,67,68]. Therefore, we here verified if AMPK activation leads to suppression of the inflammasome activation by reducing ER stress in gAcrp-stimulated breast cancer cells and found that gAcrp downregulates PERK arm of the unfolded protein response (UPR) in ER stress cascade. In addition, TUDCA and tunicamycin, classical regulators of endoplasmic reticulum homeostasis, showed exactly opposite effects on the inflammasome activation in breast cancer cells. Therefore, it appears that ER stress alleviation mediated by SESN2/AMPK axis is, at least in part, responsible for inflammasome modulation by adiponectin. However, at this stage, the molecular mechanisms by which ER stress modulates inflammasomes remain to be elucidated and further studies are required for gaining further insights.

## 4. Materials and Methods

### 4.1. Materials

All the cell culture reagents were provided by HyClone Laboratories (South Logan, UT, USA). Recombinant human globular adiponectin (gAcrp) was purchased from PeproTech Inc. (Rocky Hill, NJ, USA). Tauroursodeoxycholate (TUDCA) was obtained from MedChemExpress (Monmouth Junction, NJ, USA). MCC950, a small-molecule inhibitor of the NLRP3, was purchased from Enzo life sciences (Farmingdale, NY, USA). Compound C, a pharmacological inhibitor of AMPK, was obtained from Tocris Bioscience (Tocris House, IO Centre, Bristol, UK). Ac-YVAD-cmk and Interleukin-1 receptor antagonist (IL-1Ra) were procured from Sigma Aldrich (St Louis, MO, USA). Primary antibodies against phospho-PERK, phospho- and total eIF2α, phospho- and total AMPK, Bax, Bcl2, p53, p27 Kip1, IL-1β, and caspase-1 were purchased from Cell Signaling Technology Inc (Beverly, MA, USA); cyclin D1 and CHOP were obtained from Santa Cruz Biotechnology (Dallas, TX, USA); β-actin and total PERK were acquired from Thermo Scientific (Waltham, MA, USA); sestrin2 (SESN2) and Ki67 were provided by Abcam (Cambridge, MA, USA); and NLRP3 and ASC were procured from R&D Systems (Minneapolis, MN, USA) and Adipogen Life Sciences (San Diego, CA, USA), respectively. The secondary antibodies conjugated with horseradish peroxidase (HRP) were obtained from Thermo scientific (Waltham, MA, USA). The secondary antibodies conjugated with biotin were procured from Vector Laboratories Inc. (Burlingame, CA, USA).

### 4.2. Cell Culture

HepG2, MCF-7, MDA-MB-231, and T47D cell lines were purchased from the American Type Culture Collection (ATCC, Rockville, MD, USA). HepG2 and MCF-7 cells were maintained in Dulbecco’s modified Eagle’s medium (DMEM) containing 10% FBS and 1% penicillin–streptomycin supplemented with or without 0.1% amphotericin B, respectively. MDA-MB-231 and T47D cells were cultured in RPMI 1640 supplemented with 10% FBS and 1% penicillin–streptomycin. Cells were routinely cultured in an incubator at 37 °C under a humidified atmosphere of 95% O_2_ and 5% CO_2_.

### 4.3. Cell Viability Assay

Cell viability was determined by MTS assay as described previously [69]. Briefly, cells were seeded in 96-well plates at a density of 2 × 10^4^ cells per well. After overnight incubation, the cells were subjected to the indicated treatments in a serum-free medium followed by further incubation with 20 µL of 20 µl of 3-(4, 5-dimethylthiazol-2-yl)-5-(3-carboxymethoxyphenyl)-2-(4-sulfopheny)-2H-tetrazolium (MTS) reagent (Promega Corporation, Madison, WI, USA) for 2 h at 37 °C. The viable cells were counted by measuring the absorbance of the resultant formazan dye at 490 nm using the SPECTROstar^Nano^ microplate reader from BMG Labtech Inc (Allmendgrün, Ortenberg, Germany).

### 4.4. Caspase-7 Enzyme Activity Assay

Caspase-7 enzyme activity was measured using a Caspase-Glo 3/7 assay kit (Promega Corporation) according to the manufacturer’s instructions. In brief, cells were seeded in 96 well plates at a density of 2 × 10^4^ cells per well. After overnight incubation, cells were treated with gAcrp or inhibitors as indicated. Caspase-7 activity was determined via measurement of the luminescence emitted by the cleavage of luminogenic substrate Ac-DEVD-pNA using a micro-plate reader (Flurostar Optima, BMG Labtech, Ortenberg, Germany).

### 4.5. Cell Cycle Analysis

The cell cycle phase distribution was assessed using a Cycle test Plus DNA reagent kit (BD Biosciences, San Jose, CA, USA) according to the manufacturer’s instructions. Briefly, cells were seeded at a density of 2 × 10^5^ cells per 35 mm dish. After treatments as indicated, the cells were collected in a citrate buffer, followed by sequential incubation with Solution A (containing trypsin to digest cell membranes and cytoskeletons), Solution B (containing trypsin inhibitor and ribonuclease A to inhibit trypsin activity and digest RNA), and Solution C (containing propidium iodide for DNA staining). Finally, the samples were subjected to flow cytometric analysis (BD FACSCalibur™) and the distribution of cells in each cell cycle phase was analyzed by FlowJo 7.6 Software (FlowJo LLC, Ashland, OR, USA). 

### 4.6. RNA Isolation, Reverse Transcription (RT) and Quantitative PCR (qPCR)

Total RNA was extracted using Qiagen lysis reagent (Qiagen, Germantown, MD, USA) according to the manufacturer’s instructions. One microgram of total RNA was reverse transcribed using the Go Script reverse transcription system (Promega Corporation, Madison, WI, USA). Quantitative real-time PCR amplification was then carried out with a Roche LightCycler 2.0 (Mannheim, Germany) using the absolute qPCR SYBR green capillary mix AB gene system (Thermo scientific, UK) at 95 °C for 15 min followed by 40 cycles at 95 °C for 15 s, 60 °C for 30 s, and 72 °C for 30 s. The primer sequences used for amplification of target genes were as follows; *SESN2*: Forward 5′-CCTTCTCCACACCCAGACAT-3′, Reverse 5′-GTGCATGGCGATGGTGTTAT-3′; *pro-IL-1β*: Forward 5′-GCCCTAAACAGATGAAGTGCTC-3′, Reverse 5′-GAACCAGCATCTTCCTCAG-3′; GAPDH: Forward 5′-ACCACAGTCCATGCCATCAC-3′, Reverse 5′-TCCACCACCCTGTTGCTGTA-3′.

### 4.7. Western blot Analysis

Cells were seeded at a density of 5 × 10^5^ cells per 35 mm dish. After the indicated treatments, whole cell lysates were prepared using radio-immunoprecipitation assay lysis buffer (RIPA) containing a halt protease and phosphatase inhibitor cocktail. Equal amounts (30–50 µg) of protein were resolved on a 7.5–15% sodium dodecyl sulfate polyacrylamide gel and transferred to polyvinylidene difluoride (PVDF) membranes. For reducing non-specific binding, membranes were blocked with 5% skim milk prior to overnight incubation with the primary antibody. An appropriate secondary antibody conjugated with horseradish peroxidase (HRP) was applied to the membrane for 1 h at room temperature. Immunodetection was carried out using an enhanced chemiluminescence (ECL) detection system and images were captured by the Fujifilm LAS-4000 mini (Fujifilm, Tokyo, Japan). The whole blots can be found in supplementary (Figure S3)

### 4.8. Transient Transfection with Small Interfering RNA (siRNA)

Cells were seeded in 35 mm dishes at a density of 3 × 10^5^ cells/dish. After overnight incubation, siRNAs targeting the specific gene or scrambled control siRNA were transfected into the cells using HiPerFect Transfection Reagent (Qiagen, Hilden, Germany) according to the manufacturer’s guidelines. The gene silencing efficiency was monitored by Western blot analysis at 48 h after transfection. The siRNA duplexes used in this study were synthesized by Bioneer (Daejeon, South Korea) with the sequence as follows; *SESN2*: Forward 5′-CUGUUGCCCGAAUCCUAGU-3′, Reverse 5′-ACUAGGAUUCGGGCAACAG-3′; AMPK: Forward 5′-CUGAGUUGCAUAUACUGUA-3′, Reverse 5′-UACAGUAUAUGCAACUCAG-3′.

### 4.9. Immunoprecipitation Assay

MCF-7 cells were seeded at a density of 3 × 10^6^ cells per 100 mm dish. Total proteins were extracted with immunoprecipitation lysis buffer and incubated with 30 μL of Pierce Protein G Agarose (Thermo Scientific, Rockford, IL, USA) for 1 h at 4 °C in a rotatory mixer. The supernatants were then collected from the reaction mixture and subjected to protein quantification. The protein (500 µg) from supernatants was incubated with an appropriate primary antibody for 16 h with gentle shaking and rocking at 4 °C. The immune complexes were then pulled-down by further incubation with 30 μL of Pierce Protein G Agarose for 4 h. The beads containing immune complexes were collected and washed with immunoprecipitation lysis buffer. The protein of interest was finally released by sodium dodecyl sulphate (SDS) denaturing buffer and heating (95 °C for 10 min). The pellets were removed by centrifugation and the protein samples were stored until required for further analysis.

### 4.10. Immunocytochemistry and Immunohistochemistry

Immunocytochemistry (ICC) for the determination of ASC speck formation was performed as described previously [70]. Briefly, cells were seeded in 8-well glass chamber slides at a density of 5 × 10^4^ cells/well. After treatments as indicated, the cells were fixed with 4% paraformaldehyde, permeabilized with 0.2% Triton X-100, and blocked with 3% bovine serum albumin, followed by sequential incubation with a primary antibody specific for ASC and the fluorescein isothiocyanate (FITC)-conjugated anti-rabbit secondary antibody. Cells were then counterstained with DAPI before the images were captured with fluorescence microscopy (Nikon, Tokyo, Japan).

For immunohistochemistry analysis (IHC), tumor tissues obtained from each group were fixed with 4% paraformaldehyde before making tumor sections (30 μm) using a freezing sliding microtome (Microm HM 450, Thermo Scientific). The sections were incubated with 3% hydrogen peroxide for 20 min to quench endogenous peroxidase activity followed by rinsing and overnight incubation with a primary antibody. The sections were further incubated with an appropriate biotinylated secondary antibody for 2 h and immersed in avidin–biotin–peroxidase solution (Vector Laboratories Inc., Burlingame, CA, USA) for 1 h at room temperature. Finally, the immunocomplexes were detected by treatment with diaminobenzidine and their nuclei were counterstained with hematoxylin. The IHC images were processed using a light microscope (BX41 TF, Olympus, Tokyo, Japan). For quantification, the open-source imaging software Image J was used in combination with plugin IHC Profiler as previously described [71].

### 4.11. Development of MCF-7 Tumor Xenograft Model

All the animal studies were carried out in accordance with the guidelines of the Yeungnam University Institutional Animal Care and Use Committee (IACUC). The experimental protocols were reviewed and approved by Yeungnam University IACUC (approval date: Apr. 17, 2019 and approval number: 2019-017,). Tumor xenografts were generated by injecting 10^7^ MCF-7 cells mixed with matrigel into the rear flanks of 4 weeks old male BALB/c nude mice (Orient Ltd., Osan, South Korea). After 3 weeks of tumor implantation, mice were randomly allocated into 4 groups (*n* = 5 in each group) and received one of the following treatments: gAcrp (2 µg/mouse), MCC950 (40 µg/mouse), IL-1Ra (2 µg/mouse), and phosphate-buffered saline (served as control group). All treatments were given by intratumoral administration with a final volume of 50 µL/injection every 48 h. Tumor size was measured twice a week using digital Vernier caliper, and tumor volume (*V*) was defined as *V* =(width)^2^ × length/2. Tumor tissues were then collected after 3 weeks of treatment followed by measuring, weighing, and subjecting to further analysis.

### 4.12. Statistical Analysis

All statistical analyses were conducted with Graphpad Prism software version 5.01 and data are expressed as the mean ± standard error of the mean (SEM) from at least three independent experiments. Statistical differences among groups were analyzed using one-way analysis of variance (ANOVA), followed by Tukey’s post hoc multiple comparison tests. A *p*-value < 0.05 denotes a statistically significant difference. 

## 5. Conclusions

**Figure 7:**
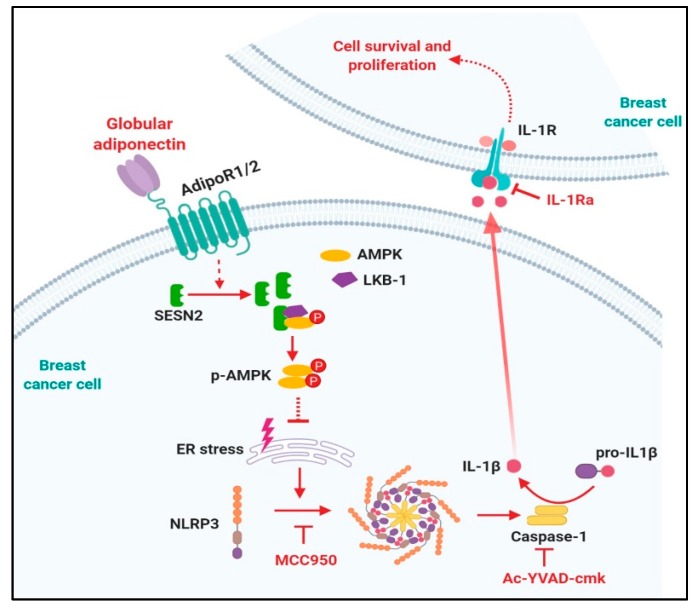
Proposed model for the modulation of NOD-like receptor pyrin domain-containing protein 3 (NLRP3) inflammasomes activation by globular adiponectin in breast cancer cells and its role in the suppression of tumor growth. Inflammasomes modulate tumor growth by complicated manners and its physiological roles would be depending on specific context. Herein, we clearly demonstrated that inflammasomes activation contributes to growth of breast cancer cells via activation of cell cycle and suppression of apoptosis. The physiological actions of adiponectin are initiated by binding with its specific transmembrane receptor (adiponectin receptor type 1 and type 2 (adipoR1 and adipoR2)). Binding of globular adiponectin with adipoR1/R2 generates signaling, which suppresses NLRP3 inflammasomes activation leading to the prevention of interleukin-1β (IL-1β) maturation and caspase-1 activation in both in vitro and in vivo xenograft models, implying that suppression of tumor growth by globular adiponectin is mediated via modulation of the inflammasome. Modulation of the inflammasome by globular adiponectin is mediated by sestrin2 (SESN2)/AMP-activated protein kinase (AMPK)/Endoplasmic reticulum (ER) stress axis. SESN2 induction leads to AMPK phosphorylation by promoting association of AMPK with its upstream kinase, LKB-1. Activation of AMPK signaling plays a pivotal role in reduction in ER stress, which subsequently leads to prevention of inflammasomes activation. In here, inflammasomes activation and IL-1β signaling might be required for survival and growth of breast tumor growth. Therefore, negative modulation of inflammasomes activation by globular adiponectin, MCC950, Ac-YVAD, and IL-1 receptor antagonist promotes apoptosis and impedes cancer cell proliferation. Therefore, modulation of inflammasomes and various molecules involved would be potential therapeutic targets for the treatment of breast cancer. The molecular mechanisms underlying SESN2 induction by globular adiponectin remain elusive. In addition, the molecular mechanisms by which ER stress induces inflammasomes activation and IL-1 signaling modulates apoptosis and cell cycle remain to be elucidated.

In summary, the results presented in this study have provided the first evidence that globular adiponectin suppresses the NLRP3 inflammasome activation in breast and hepatic cancer cells. Mechanistically, inflammasomes inhibition by globular adiponectin is dependent on SESN2-promoted AMPK phosphorylation, which subsequently contributes to relieve ER stress. Moreover, we have clearly demonstrated that inflammasomes activation leads to growth of breast cancer cells by modulating both apoptosis and cell cycle arrest (Figure 7). Based on these findings, inhibition of inflammasomes might be a novel mechanism for the antitumor activity of adiponectin in breast cancer cells. In addition, taken into consideration a potential link between obesity and inflammasomes activation, as well as inverse correlation of serum adiponectin levels and risk of breast cancer, targeting the inflammasome could be a promising strategy for the treatment of breast cancer. 

## Supplementary Materials

The following are available online at https://www.mdpi.com/2072-6694/12/3/613/s1, Figure S1: Effect of globular adiponectin on SESN2 mRNA expression level in breast cancer cells, Figure S2: Effects of inflammasomes inhibitors on the viability of breast cancer cells, Figure S3: Blots from Western blot analysis.

## References

[1] Divella R., De Luca R., Abbate I., Naglieri E., Daniele A. (2016). Obesity and cancer: The role of adipose tissue and adipo-cytokines-induced chronic inflammation. J. Cancer.

[2] Rajala M.W., Scherer P.E. (2003). Minireview: The adipocyte—At the crossroads of energy homeostasis, inflammation, and atherosclerosis. Endocrinology.

[3] Waki H., Yamauchi T., Kamon J., Kita S., Ito Y., Hada Y., Uchida S., Tsuchida A., Takekawa S., Kadowaki T. (2005). Generation of globular fragment of adiponectin by leukocyte elastase secreted by monocytic cell line thp-1. Endocrinology.

[4] Tilg H., Moschen A.R. (2006). Adipocytokines: Mediators linking adipose tissue, inflammation and immunity. Nat. Rev. Immunol..

[5] Fruebis J., Tsao T.S., Javorschi S., Ebbets-Reed D., Erickson M.R., Yen F.T., Bihain B.E., Lodish H.F. (2001). Proteolytic cleavage product of 30-kda adipocyte complement-related protein increases fatty acid oxidation in muscle and causes weight loss in mice. Proc. Natl. Acad. Sci. USA.

[6] Berg A.H., Combs T.P., Scherer P.E. (2002). Acrp30/adiponectin: An adipokine regulating glucose and lipid metabolism. Trends Endocrinol. Metab..

[7] Grossmann M.E., Nkhata K.J., Mizuno N.K., Ray A., Cleary M.P. (2008). Effects of adiponectin on breast cancer cell growth and signaling. Br. J. Cancer.

[8] Katira A., Tan P.H. (2016). Evolving role of adiponectin in cancer-controversies and update. Cancer Biol. Med..

[9] Dalamaga M., Diakopoulos K.N., Mantzoros C.S. (2012). The role of adiponectin in cancer: A review of current evidence. Endocr. Rev..

[10] Danthala M., Rajesh G., Gundeti S., Raju G., Chandran P., Srinivas M. (2018). Obesity and breast cancer: Association of serum adiponectin, leptin, and adiponectin-leptin ratio as risk biomarkers. Indian J. Med. Paediatr. Oncol..

[11] Shrestha A., Nepal S., Kim M.J., Chang J.H., Kim S.H., Jeong G.S., Jeong C.H., Park G.H., Jung S., Lim J. (2016). Critical role of ampk/foxo3a axis in globular adiponectin-induced cell cycle arrest and apoptosis in cancer cells. J. Cell. Physiol..

[12] Cui E., Guo H., Shen M., Yu H., Gu D., Mao W., Wang X. (2018). Adiponectin inhibits migration and invasion by reversing epithelial-mesenchymal transition in non-small cell lung carcinoma. Oncol. Rep..

[13] Nigro E., Schettino P., Polito R., Scudiero O., Monaco M.L., De Palma G.D., Daniele A. (2018). Adiponectin and colon cancer: Evidence for inhibitory effects on viability and migration of human colorectal cell lines. Mol. Cell. Biochem..

[14] Park P.H. (2018). Autophagy induction: A critical event for the modulation of cell death/survival and inflammatory responses by adipokines. Arch. Pharm. Res..

[15] Chung S.J., Nagaraju G.P., Nagalingam A., Muniraj N., Kuppusamy P., Walker A., Woo J., Gyorffy B., Gabrielson E., Saxena N.K. (2017). Adipoq/adiponectin induces cytotoxic autophagy in breast cancer cells through stk11/lkb1-mediated activation of the ampk-ulk1 axis. Autophagy.

[16] Xu S., Li X., Liu Y., Xia Y., Chang R., Zhang C. (2019). Inflammasome inhibitors: Promising therapeutic approaches against cancer. J. Hematol. Oncol..

[17] Broz P., Dixit V.M. (2016). Inflammasomes: Mechanism of assembly, regulation and signalling. Nat. Rev. Immunol..

[18] Kong H., Wang Y., Zeng X., Wang Z., Wang H., Xie W. (2015). Differential expression of inflammasomes in lung cancer cell lines and tissues. Tumour Biol..

[19] Zhai Z., Liu W., Kaur M., Luo Y., Domenico J., Samson J.M., Shellman Y.G., Norris D.A., Dinarello C.A., Spritz R.A. (2017). Nlrp1 promotes tumor growth by enhancing inflammasome activation and suppressing apoptosis in metastatic melanoma. Oncogene.

[20] Wang H., Luo Q., Feng X., Zhang R., Li J., Chen F. (2018). Nlrp3 promotes tumor growth and metastasis in human oral squamous cell carcinoma. BMC Cancer.

[21] Ershaid N., Sharon Y., Doron H., Raz Y., Shani O., Cohen N., Monteran L., Leider-Trejo L., Ben-Shmuel A., Yassin M. (2019). Nlrp3 inflammasome in fibroblasts links tissue damage with inflammation in breast cancer progression and metastasis. Nat. Commun..

[22] Kolb R., Phan L., Borcherding N., Liu Y., Yuan F., Janowski A.M., Xie Q., Markan K.R., Li W., Potthoff M.J. (2016). Obesity-associated nlrc4 inflammasome activation drives breast cancer progression. Nat. Commun..

[23] Kantono M., Guo B. (2017). Inflammasomes and cancer: The dynamic role of the inflammasome in tumor development. Front. Immunol..

[24] Karki R., Man S.M., Kanneganti T.-D. (2017). Inflammasomes and cancer. Cancer Immunol. Res..

[25] Yu J., Li S., Qi J., Chen Z., Wu Y., Guo J., Wang K., Sun X., Zheng J. (2019). Cleavage of gsdme by caspase-3 determines lobaplatin-induced pyroptosis in colon cancer cells. Cell Death Dis..

[26] Zhang C.-C., Li C.-G., Wang Y.-F., Xu L.-H., He X.-H., Zeng Q.-Z., Zeng C.-Y., Mai F.-Y., Hu B., Ouyang D.-Y. (2019). Chemotherapeutic paclitaxel and cisplatin differentially induce pyroptosis in a549 lung cancer cells via caspase-3/gsdme activation. Apoptosis.

[27] Pizato N., Luzete B.C., Kiffer L.F.M.V., Corrêa L.H., de Oliveira Santos I., Assumpção J.A.F., Ito M.K., Magalhães K.G. (2018). Omega-3 docosahexaenoic acid induces pyroptosis cell death in triple-negative breast cancer cells. Sci. Rep..

[28] Kim M.J., Kim E.H., Pun N.T., Chang J.H., Kim J.A., Jeong J.H., Choi D.Y., Kim S.H., Park P.H. (2017). Globular adiponectin inhibits lipopolysaccharide-primed inflammasomes activation in macrophages via autophagy induction: The critical role of ampk signaling. Int. J. Mol. Sci..

[29] Wang F., Liu Y., Yang W., Yuan J., Mo J. (2018). Adiponectin inhibitor nlrp3 inflammasome by modulating the ampk-ros pathway. Int. J. Clin. Exp. Pathol..

[30] Ho A., Cho C.S., Namkoong S., Cho U.S., Lee J.H. (2016). Biochemical basis of sestrin physiological activities. Trends Biochem. Sci..

[31] Zhang X.Y., Wu X.Q., Deng R., Sun T., Feng G.K., Zhu X.F. (2013). Upregulation of sestrin 2 expression via jnk pathway activation contributes to autophagy induction in cancer cells. Cell. Signal..

[32] Wei J.-L., Fang M., Fu Z.-X., Zhang S.-R., Guo J.-B., Wang R., Lv Z.-B., Xiong Y.-F. (2017). Sestrin 2 suppresses cells proliferation through ampk/mtorc1 pathway activation in colorectal cancer. Oncotarget.

[33] Sanli T., Linher-Melville K., Tsakiridis T., Singh G. (2012). Sestrin2 modulates ampk subunit expression and its response to ionizing radiation in breast cancer cells. PLoS ONE.

[34] Kim M.J., Bae S.H., Ryu J.C., Kwon Y., Oh J.H., Kwon J., Moon J.S., Kim K., Miyawaki A., Lee M.G. (2016). Sesn2/sestrin2 suppresses sepsis by inducing mitophagy and inhibiting nlrp3 activation in macrophages. Autophagy.

[35] Yang F., Qin Y., Wang Y., Meng S., Xian H., Che H., Lv J., Li Y., Yu Y., Bai Y. (2019). Metformin inhibits the nlrp3 inflammasome via ampk/mtor-dependent effects in diabetic cardiomyopathy. Int. J. Biol. Sci..

[36] Cordero M.D., Williams M.R., Ryffel B. (2018). Amp-activated protein kinase regulation of the nlrp3 inflammasome during aging. Trends Endocrinol. Metab..

[37] Li Y., Li J., Li S., Li Y., Wang X., Liu B., Fu Q., Ma S. (2015). Curcumin attenuates glutamate neurotoxicity in the hippocampus by suppression of er stress-associated txnip/nlrp3 inflammasome activation in a manner dependent on ampk. Toxicol. Appl. Pharmacol..

[38] Bae H.R., Kim D.H., Park M.H., Lee B., Kim M.J., Lee E.K., Chung K.W., Kim S.M., Im D.S., Chung H.Y. (2016). Beta-hydroxybutyrate suppresses inflammasome formation by ameliorating endoplasmic reticulum stress via ampk activation. Oncotarget.

[39] Park H.W., Park H., Ro S.H., Jang I., Semple I.A., Kim D.N., Kim M., Nam M., Zhang D., Yin L. (2014). Hepatoprotective role of sestrin2 against chronic er stress. Nat. Commun..

[40] Wei Q., Guo P., Mu K., Zhang Y., Zhao W., Huai W., Qiu Y., Li T., Ma X., Liu Y. (2015). Estrogen suppresses hepatocellular carcinoma cells through erbeta-mediated upregulation of the nlrp3 inflammasome. Lab. Investig..

[41] Sharma D., Kanneganti T.-D. (2016). The cell biology of inflammasomes: Mechanisms of inflammasome activation and regulation. J. Cell Biol..

[42] Lei C., Huang C.-F., Li Y., Deng W.-W., Mao L., Wu L., Zhang W.-F., Zhang L., Zhi S. (2018). Blockage of the nlrp3 inflammasome by mcc950 improves anti-tumor immune responses in head and neck squamous cell carcinoma. Cell. Mol. Life Sci..

[43] Siterman M., Lengier S., Zadik L., Ofir N., Nachmias N. (2017). Mcc950 a novel inhibitor of nlrp3 inflammasome reduces migration and invasion of lung adenocarcinoma in-vitro. Am. J. Respir. Crit. Care Med..

[44] Sun Y., Guo Y. (2018). Expression of caspase-1 in breast cancer tissues and its effects on cell proliferation, apoptosis and invasion. Oncol. Lett..

[45] Schlosser S., Gansauge F., Ramadani M., Beger H.G., Gansauge S. (2001). Inhibition of caspase-1 induces cell death in pancreatic carcinoma cells and potentially modulates expression levels of bcl-2 family proteins. FEBS Lett..

[46] Cleary M.P., Grossmann M.E. (2009). Obesity and Breast Cancer: The Estrogen Connection. Endocrinology.

[47] Andò S., Gelsomino L., Panza S., Giordano C., Bonofiglio D., Barone I., Catalano S. (2019). Obesity, Leptin and Breast Cancer: Epidemiological Evidence and Proposed Mechanisms. Cancers (Basel).

[48] Raut P.K., Choi D.Y., Kim S.H., Hong J.T., Kwon T.K., Jeong J.H., Park P.-H. (2017). Estrogen receptor signaling mediates leptin-induced growth of breast cancer cells via autophagy induction. Oncotarget.

[49] Raut P.K., Kim S.-H., Choi D.Y., Jeong G.-S., Park P.-H. (2019). Growth of breast cancer cells by leptin is mediated via activation of the inflammasome: Critical roles of estrogen receptor signaling and reactive oxygen species production. Biochem. Pharmacol..

[50] Renehan A.G., Zwahlen M., Egger M. (2015). Adiposity and cancer risk: New mechanistic insights from epidemiology. Nat. Rev. Cancer.

[51] Iyengar N.M., Zhou X.K., Gucalp A., Morris P.G., Howe L.R., Giri D.D., Morrow M., Wang H., Pollak M., Jones L.W. (2016). Systemic correlates of white adipose tissue inflammation in early-stage breast cancer. Clin. Cancer Res..

[52] Sánchez-Jiménez F., Pérez-Pérez A., de la Cruz-Merino L., Sánchez-Margalet V. (2019). Obesity and breast cancer: Role of leptin. Front. Oncol..

[53] Ahechu P., Zozaya G., Marti P., Hernandez-Lizoain J.L., Baixauli J., Unamuno X., Fruhbeck G., Catalan V. (2018). Nlrp3 inflammasome: A possible link between obesity-associated low-grade chronic inflammation and colorectal cancer development. Front. Immunol..

[54] Harvey A.E., Lashinger L.M., Hursting S.D. (2011). The growing challenge of obesity and cancer: An inflammatory issue. Ann. N. Y. Acad. Sci..

[55] Stienstra R., Joosten L.A., Koenen T., van Tits B., van Diepen J.A., van den Berg S.A., Rensen P.C., Voshol P.J., Fantuzzi G., Hijmans A. (2010). The inflammasome-mediated caspase-1 activation controls adipocyte differentiation and insulin sensitivity. Cell Metab..

[56] Vandanmagsar B., Youm Y.-H., Ravussin A., Galgani J.E., Stadler K., Mynatt R.L., Ravussin E., Stephens J.M., Dixit V.D. (2011). The nlrp3 inflammasome instigates obesity-induced inflammation and insulin resistance. Nat. Med..

[57] Youm Y.-H., Adijiang A., Vandanmagsar B., Burk D., Ravussin A., Dixit V.D. (2011). Elimination of the nlrp3-asc inflammasome protects against chronic obesity-induced pancreatic damage. Endocrinology.

[58] Coppola A., Marfella R., Coppola L., Tagliamonte E., Fontana D., Liguori E., Cirillo T., Cafiero M., Natale S., Astarita C. (2009). Effect of weight loss on coronary circulation and adiponectin levels in obese women. Int. J. Cardiol..

[59] Li J., Han X. (2018). Adipocytokines and breast cancer. Curr. Probl. Cancer.

[60] Wolin K.Y., Carson K., Colditz G.A. (2010). Obesity and cancer. Oncologist.

[61] Ro S.H., Semple I.A., Park H., Park H.W., Kim M., Kim J.S., Lee J.H. (2014). Sestrin2 promotes unc-51-like kinase 1 mediated phosphorylation of p62/sequestosome-1. FEBS J..

[62] Chai D., Wang G., Zhou Z., Yang H., Yu Z. (2015). Insulin increases sestrin 2 content by reducing its degradation through the pi 3 k/mtor signaling pathway. Int. J. Endocrinol..

[63] Taliaferro-Smith L., Nagalingam A., Zhong D., Zhou W., Saxena N.K., Sharma D. (2009). Lkb1 is required for adiponectin-mediated modulation of ampk-s6k axis and inhibition of migration and invasion of breast cancer cells. Oncogene.

[64] Wang F., Wang L., Liu Y., Yuan J., Mo Z. (2018). Adiponectin attenuates nlrp3 inflammasome by modulating ampk-ros pathway. Diabetes.

[65] Urra H., Dufey E., Avril T., Chevet E., Hetz C. (2016). Endoplasmic reticulum stress and the hallmarks of cancer. Trends Cancer.

[66] Oakes S.A. (2017). Endoplasmic reticulum proteostasis: A key checkpoint in cancer. Am. J. Physiol. Cell Physiol..

[67] Li J., Wang Y., Wang Y., Wen X., Ma X.-N., Chen W., Huang F., Kou J., Qi L.-W., Liu B. (2015). Pharmacological activation of ampk prevents drp1-mediated mitochondrial fission and alleviates endoplasmic reticulum stress-associated endothelial dysfunction. J. Mol. Cell. Cardiol..

[68] Li A., Zhang S., Li J., Liu K., Huang F., Liu B. (2016). Metformin and resveratrol inhibit drp1-mediated mitochondrial fission and prevent er stress-associated nlrp3 inflammasome activation in the adipose tissue of diabetic mice. Mol. Cell. Endocrinol..

[69] Nepal S., Park P.-H. (2013). Activation of autophagy by globular adiponectin attenuates ethanol-induced apoptosis in hepg2 cells: Involvement of ampk/foxo3a axis. Biochim. Biophys. Acta (BBA) Mol. Cell Res..

[70] Nepal S., Shrestha A., Park P.-H. (2015). Ubiquitin specific protease 2 acts as a key modulator for the regulation of cell cycle by adiponectin and leptin in cancer cells. Mol. Cell. Endocrinol..

[71] Varghese F., Bukhari A.B., Malhotra R., De A. (2014). Ihc profiler: An open source plugin for the quantitative evaluation and automated scoring of immunohistochemistry images of human tissue samples. PLoS ONE.

