# Supplementary material for: Globular Adiponectin Inhibits Breast Cancer Cell Growth through Modulation of Inflammasome Activation: Critical Role of Sestrin2 and AMPK Signaling
Source: Cancers (Basel). 2020 Mar 6;12(3):613. doi: 10.3390/cancers12030613 (PMC7139717; doi:10.3390/cancers12030613)
Supplement: Supplementary file 1 [file cancers-12-00613-s001.pdf]

Globular Adiponectin Inhibits Breast Cancer Cell Growth through Modulation of Inflammasomes Activation: Critical Role of Sestrin2 and AMPK Signaling

Duc-Vinh Pham, Pawan Kumar Raut, Mahesh Pandit, Jae-Hoon Chang, Nikita Katila, Dong-Young Choi, Jee-Heon Jeong and Pil-Hoon Park

Supplementary Materials

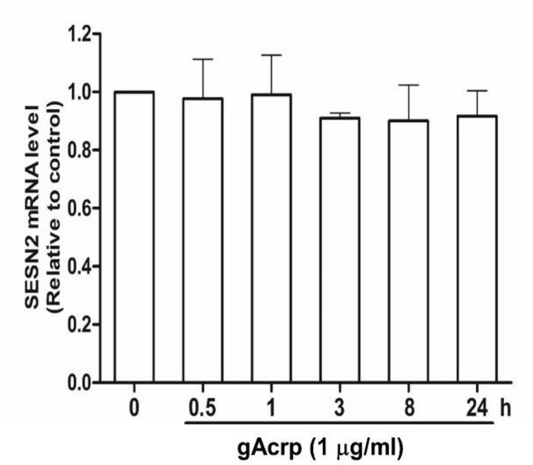

Figure S1. Effect of globular adiponectin on SESN2 mRNA expression level in breast cancer cells. MCF-7 cells were treated with gAcrp (1 μg/mL) for indicated time periods. Sestrin2 mRNA levels were determined by qT-PCR analysis.

Figure S2

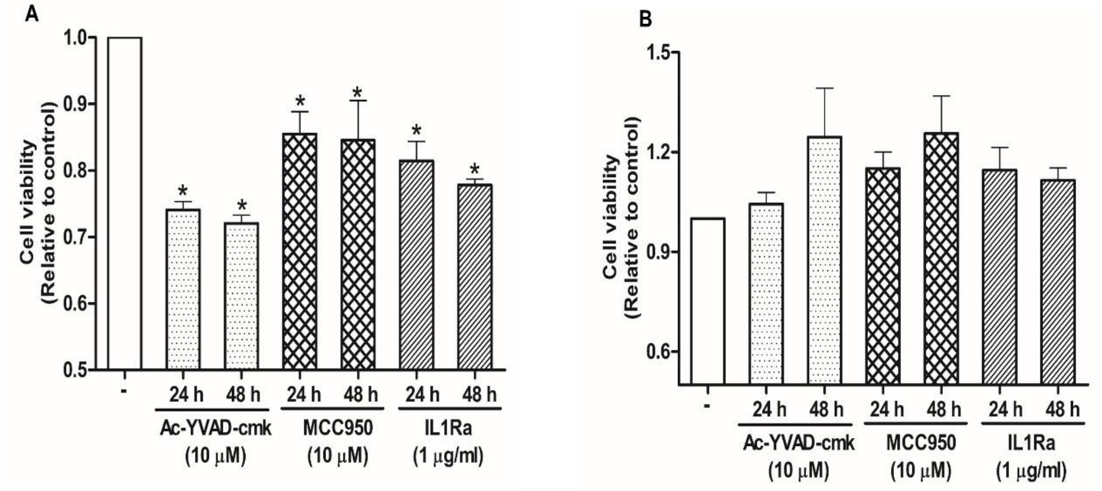

Figure S2. Figure S2. Effect of inflammasomes inhibitors on the viability of breast cancer cells. T47D cells (A) or MDA-MB-231 cells (B) were treated with Ac-YVAD-cmk, MCC950, or IL-1Ra for indicated time durations. Cell viability was determined by MTS assay.

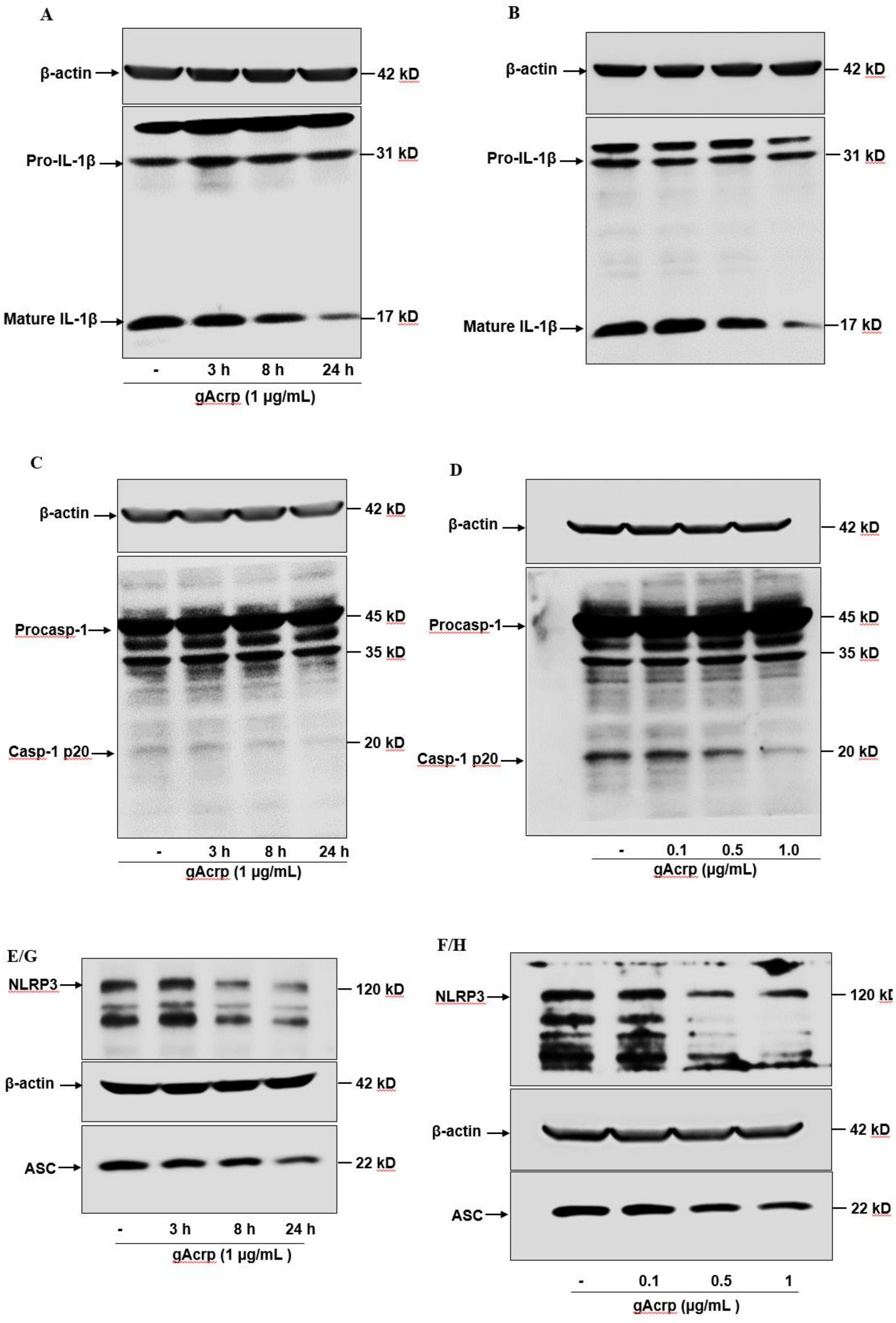
A
β-actin
42 kD
Pro-IL-1β
31 kD
Mature IL-1β
17 kD
- 3 h 8 h 24 h
gAcrp (1 μg/mL)
B
β-actin
42 kD
Pro-IL-1β
31 kD
Mature IL-1β
17 kD
C
β-actin
42 kD
Procasp-1
45 kD
35 kD
Casp-1 p20
20 kD
- 3 h 8 h 24 h
gAcrp (1 μg/mL)
D
β-actin
42 kD
Procasp-1
45 kD
35 kD
Casp-1 p20
20 kD
- 0.1 0.5 1.0
gAcrp (μg/mL)
E/G
NLRP3
120 kD
β-actin
42 kD
ASC
22 kD
- 3 h 8 h 24 h
gAcrp (1 μg/mL)
F/H
NLRP3
120 kD
β-actin
42 kD
ASC
22 kD
- 0.1 0.5 1
gAcrp (μg/mL)

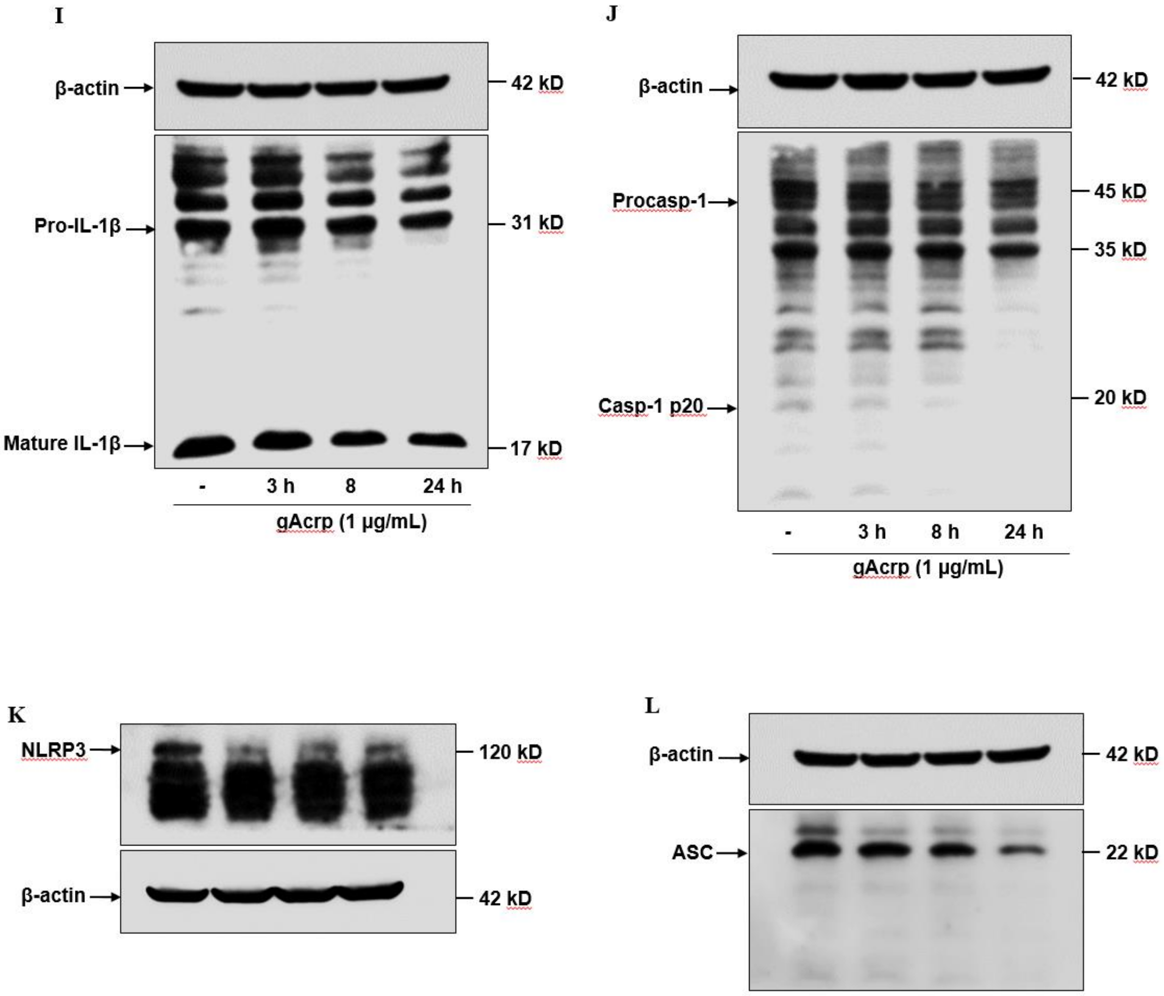
I
β-actin
42 kD
Pro-IL-1β
31 kD
Mature IL-1β
17 kD
-
3 h
8
24 h
gAcrp (1 μg/mL)
J
β-actin
42 kD
Procasp-1
45 kD
35 kD
Casp-1 p20
20 kD
-
3 h
8 h
24 h
gAcrp (1 μg/mL)
K
NLRP3
120 kD
β-actin
42 kD
L
β-actin
42 kD
ASC
22 kD

Whole Western blots for Figure 2

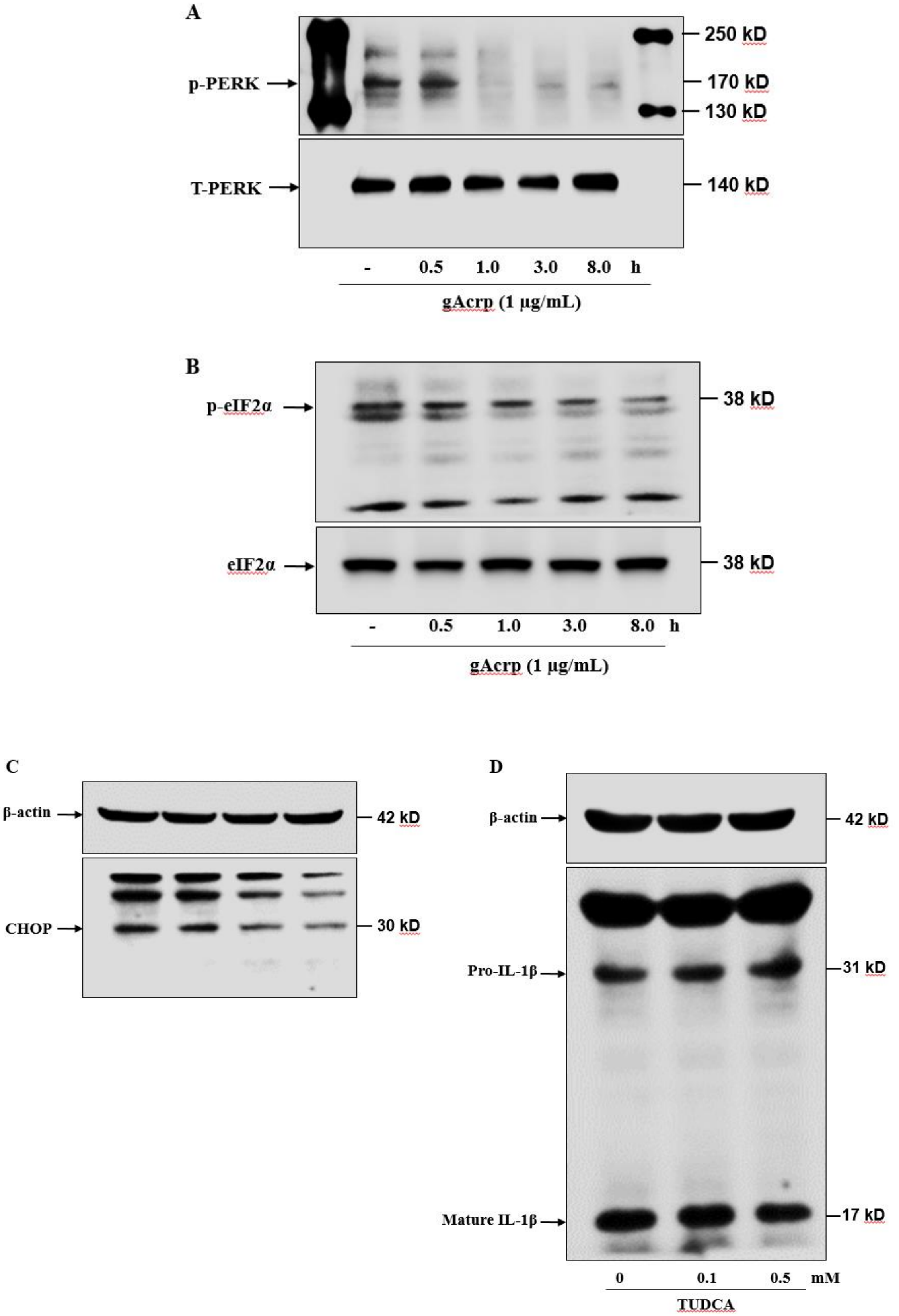

E

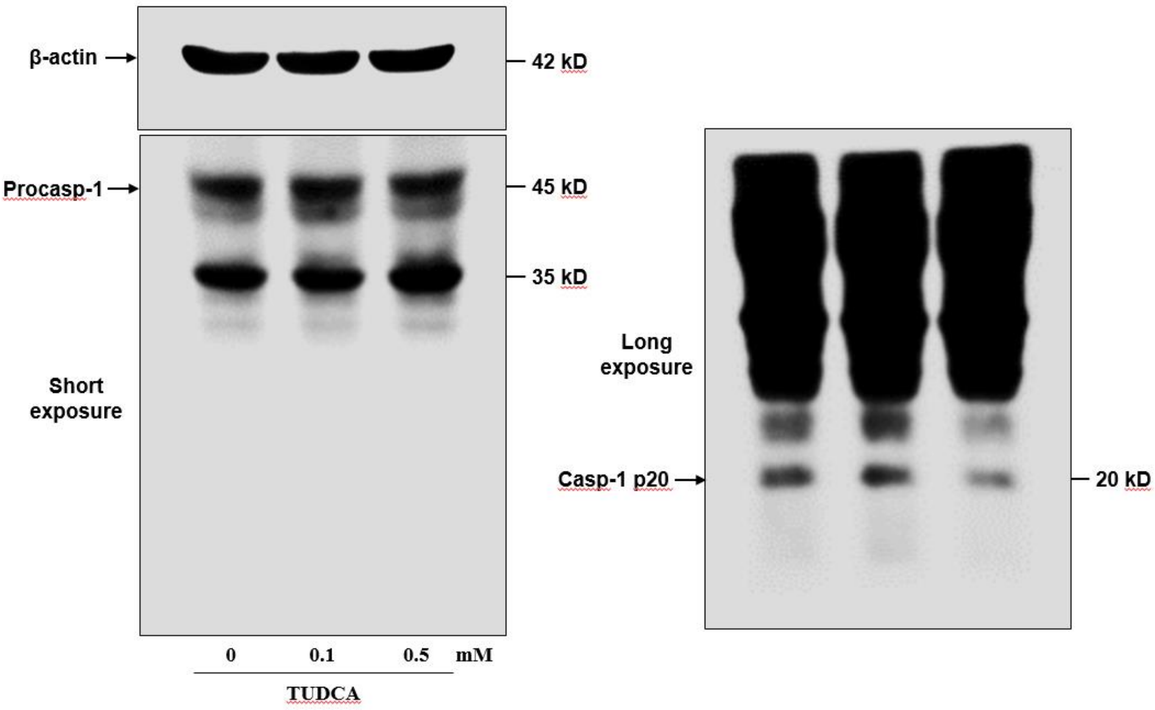
β-actin
42 kD
Procasp-1
45 kD
35 kD
Short exposure
Long exposure
Casp-1 p20
20 kD
0
0.1
0.5
mM
TUDCA

F

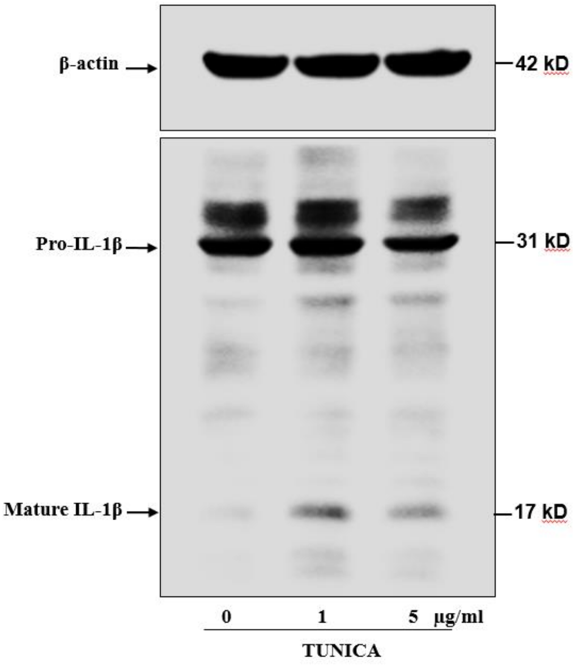
β-actin
42 kD
Pro-IL-1β
31 kD
Mature IL-1β
17 kD
0
1
5
µg/ml
TUNICA

G

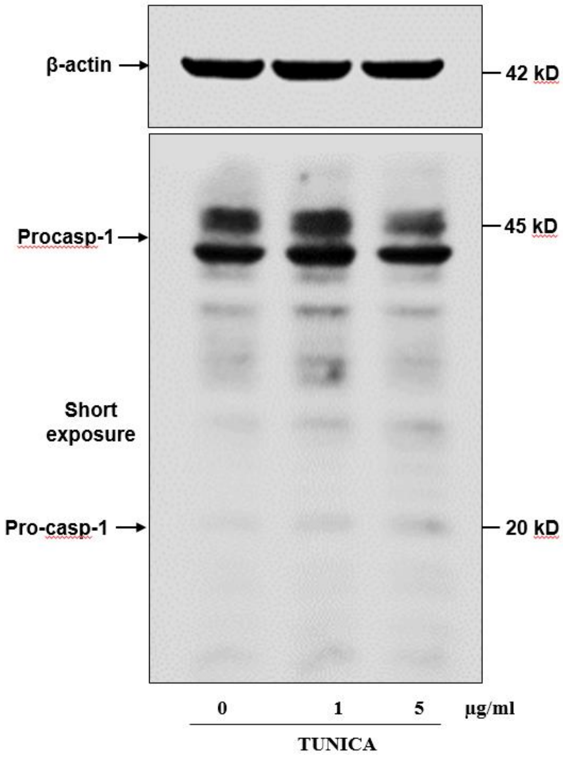
β-actin
42 kD
Procasp-1
45 kD
Short exposure
Pro-casp-1
20 kD
0
1
5
µg/ml
TUNICA

Whole Western blots for Figure 3

A

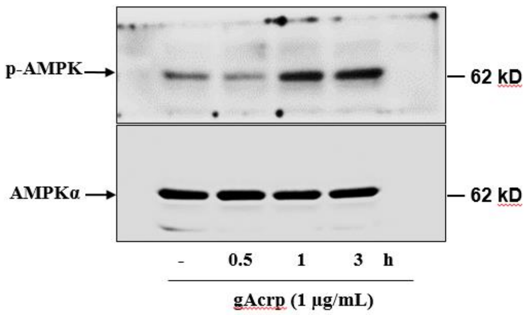

B

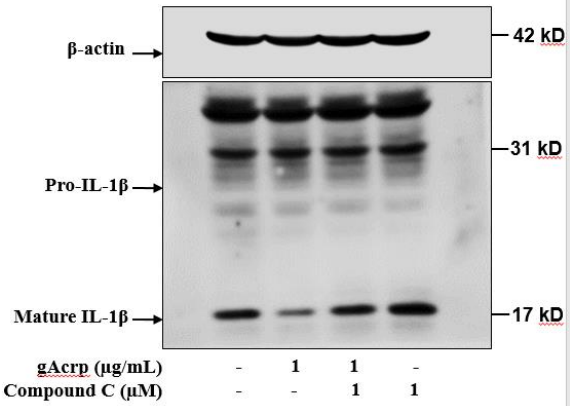

C

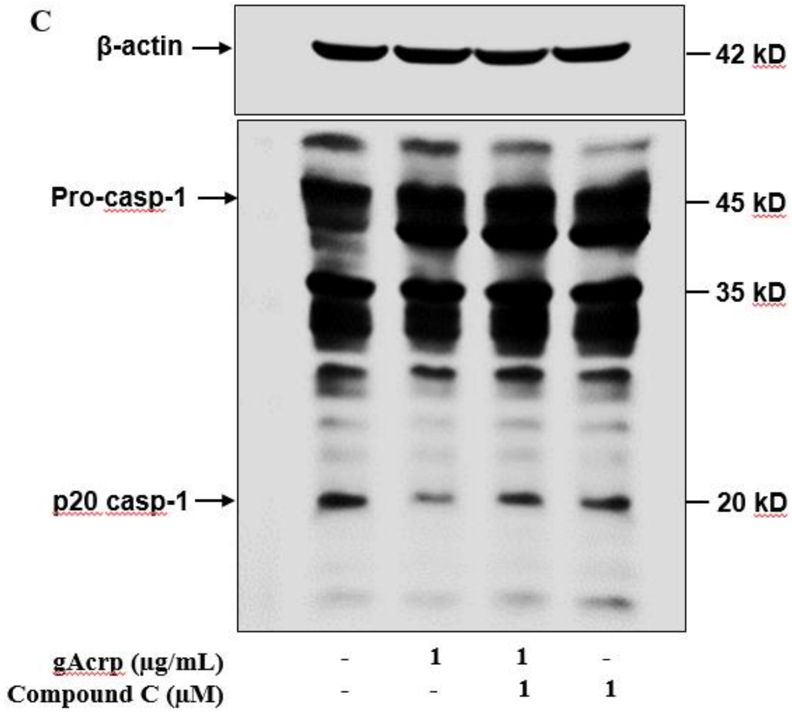

E

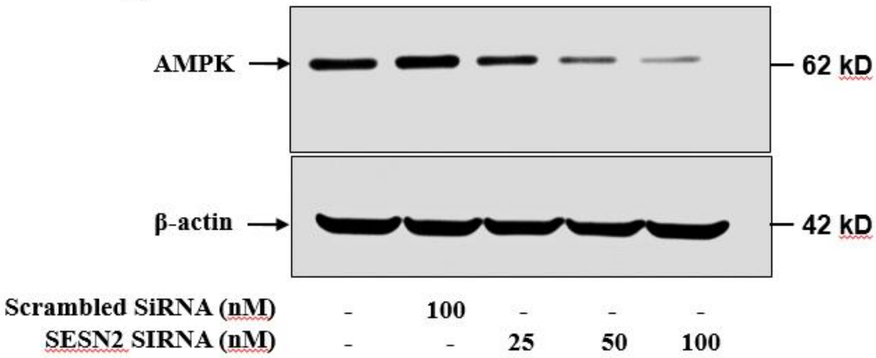

E

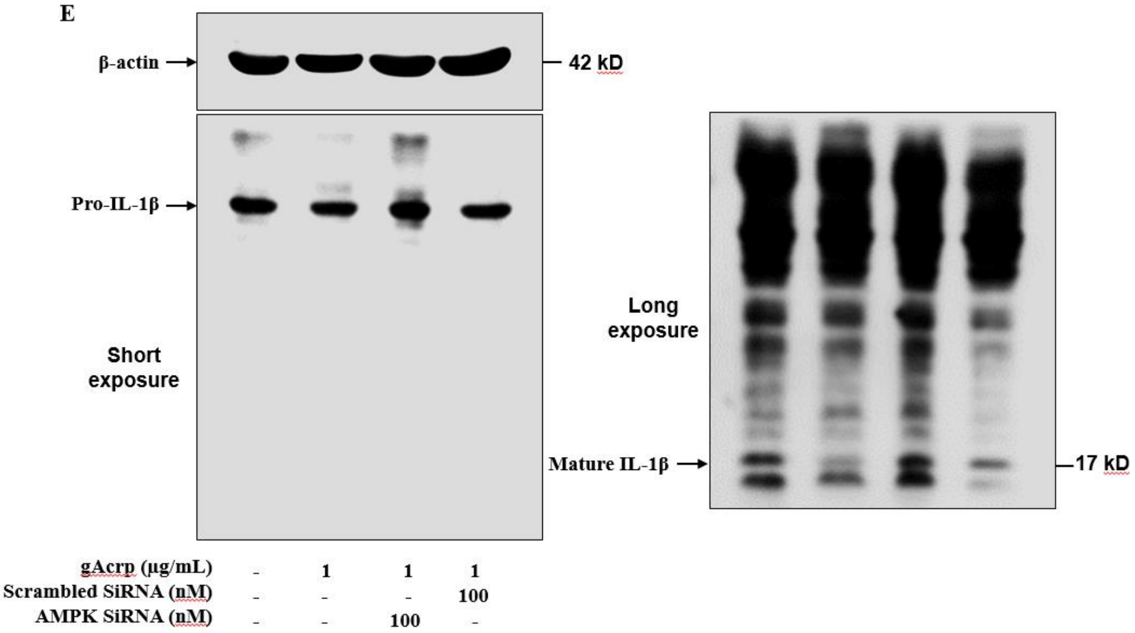
β-actin
42 kD
Pro-IL-1β
Short exposure
Long exposure
Mature IL-1β
17 kD
gAcrp (μg/mL) - 1 1 1
Scrambled SiRNA (nM) - - - 100
AMPK SiRNA (nM) - - 100 -

F

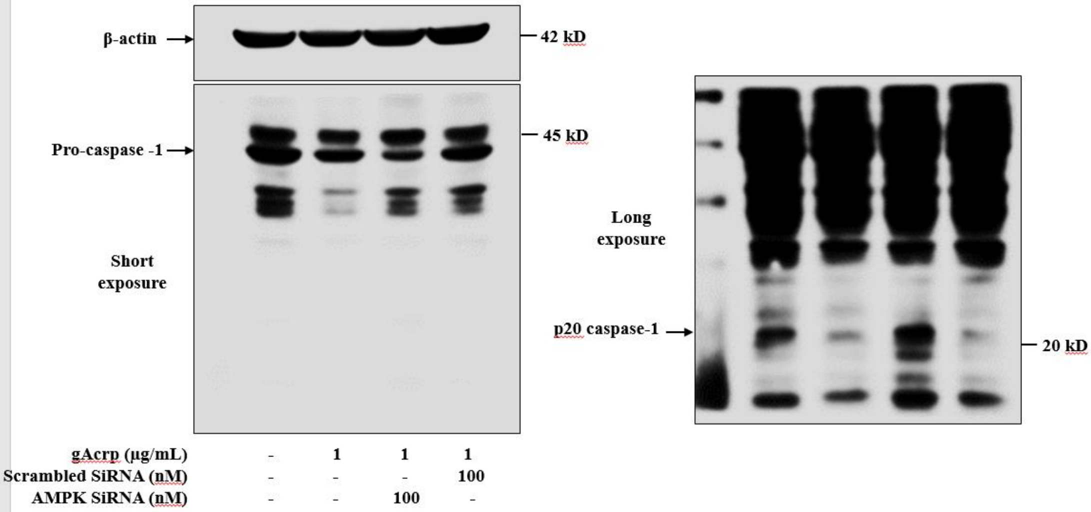
β-actin
42 kD
Pro-caspase -1
45 kD
Short exposure
Long exposure
p20 caspase-1
20 kD
gAcrp (μg/mL) - 1 1 1
Scrambled SiRNA (nM) - - - 100
AMPK SiRNA (nM) - - 100 -

G

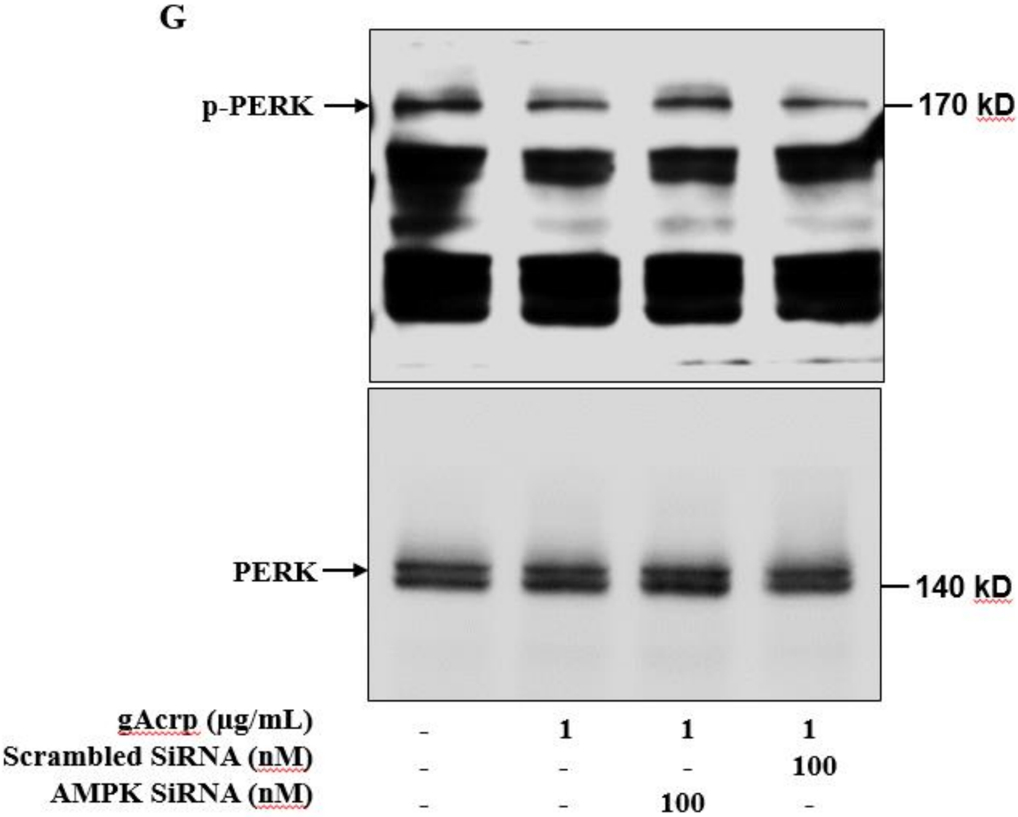
p-PERK
170 kD
PERK
140 kD
gAcrp (µg/mL) - 1 1 1
Scrambled SiRNA (nM) - - - 100
AMPK SiRNA (nM) - - 100 -

H

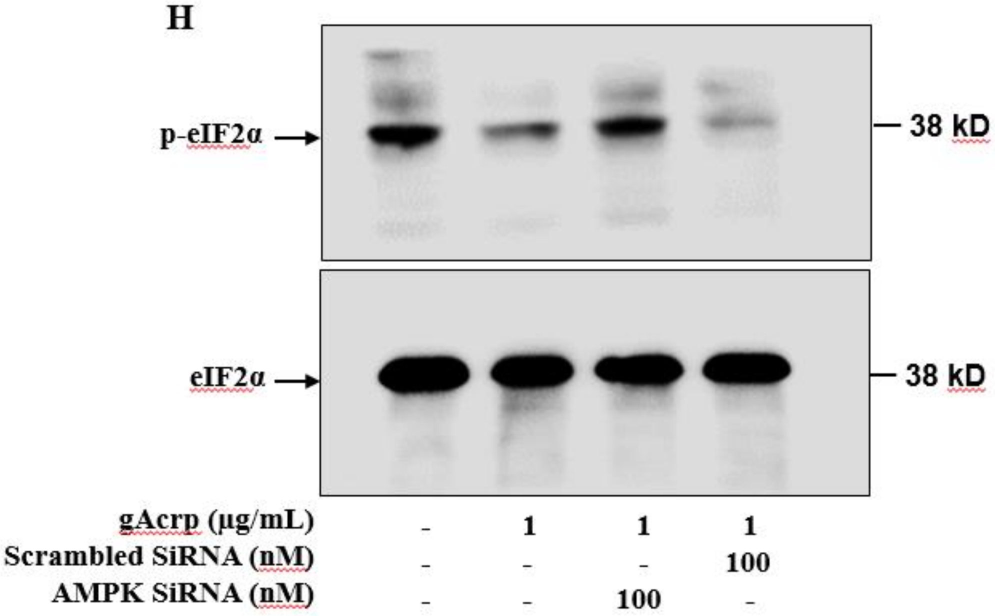
p-eIF2α
eIF2α
38 kD
38 kD
gAcrp (µg/mL) - 1 1 1
Scrambled SiRNA (nM) - - - 100
AMPK SiRNA (nM) - - 100 -

I

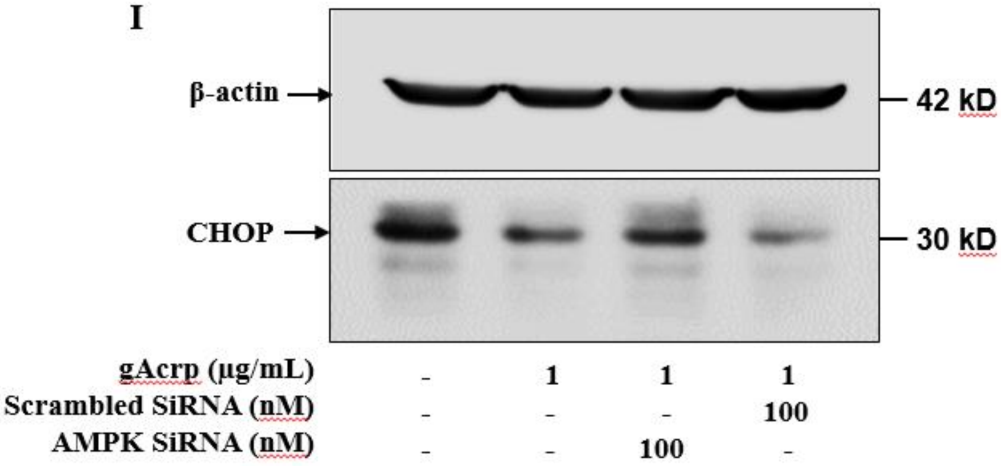
β-actin
CHOP
42 kD
30 kD
gAcrp (µg/mL) - 1 1 1
Scrambled SiRNA (nM) - - - 100
AMPK SiRNA (nM) - - 100 -

Whole Western blots for Figure 4

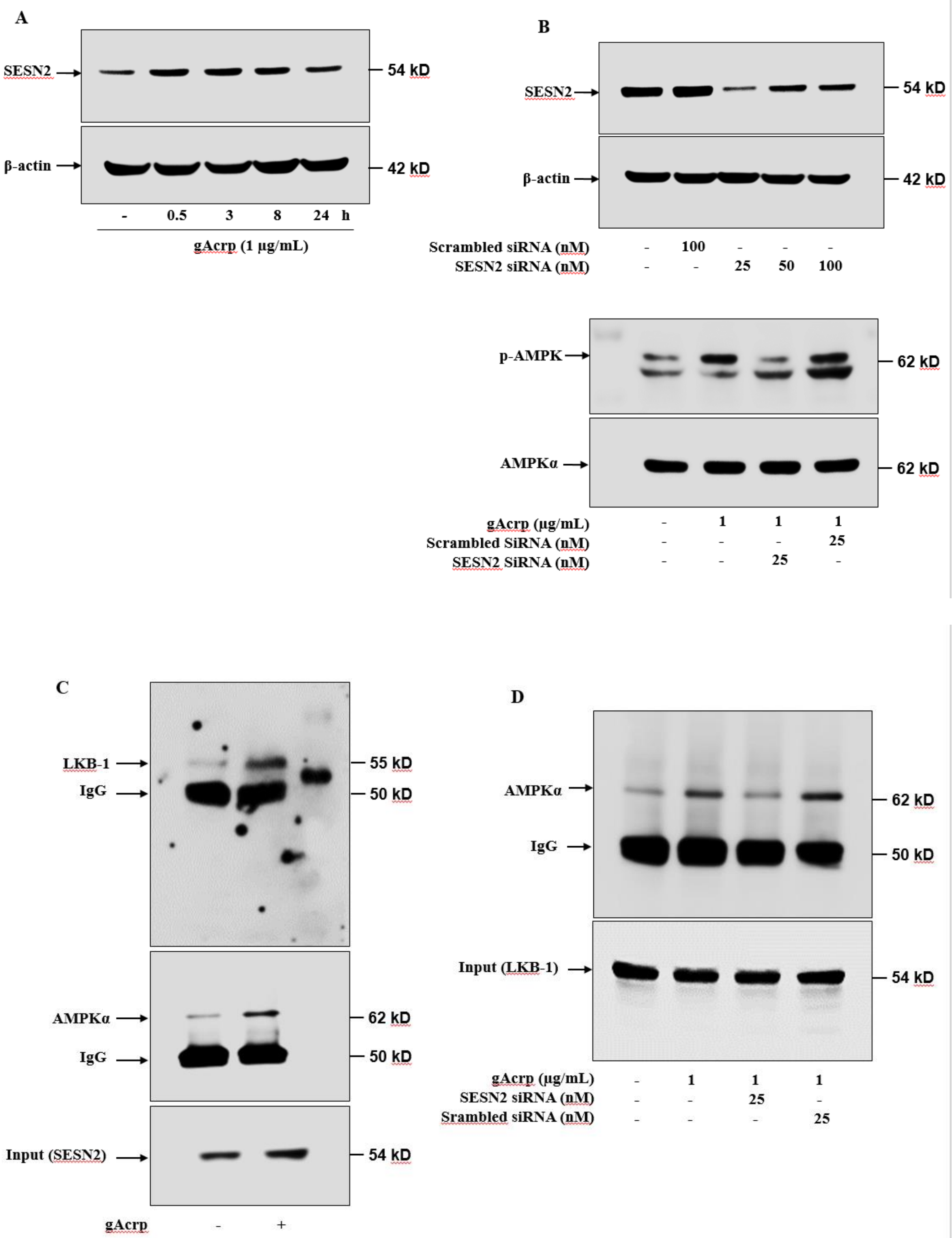

E

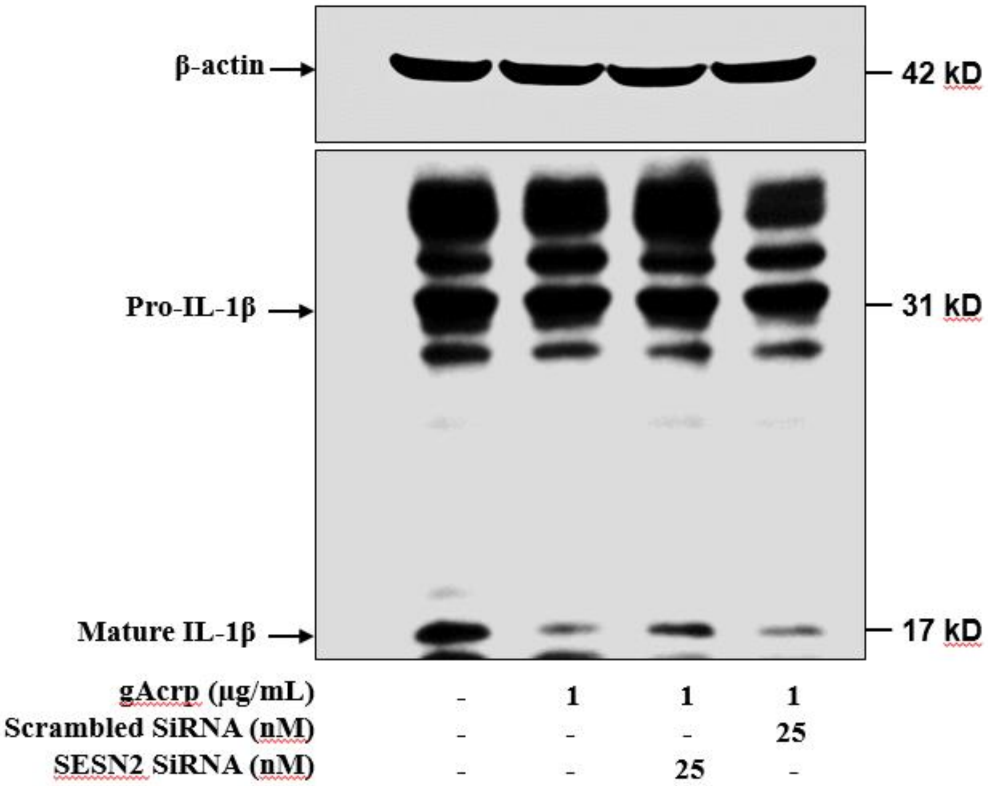
β-actin
42 kD
Pro-IL-1β
31 kD
Mature IL-1β
17 kD
gAcrp (µg/mL) - 1 1 1
Scrambled SiRNA (nM) - - - 25
SESN2 SiRNA (nM) - - 25 -

F

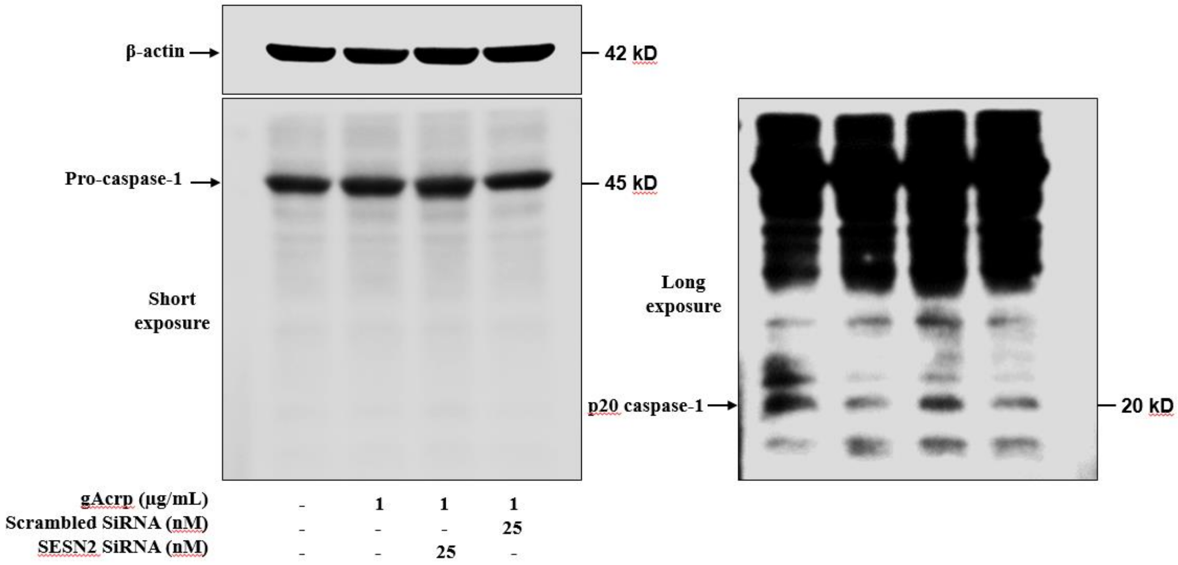
β-actin
42 kD
Pro-caspase-1
45 kD
Short exposure
Long exposure
p20 caspase-1
20 kD
gAcrp (µg/mL) - 1 1 1
Scrambled SiRNA (nM) - - - 25
SESN2 SiRNA (nM) - - 25 -

G

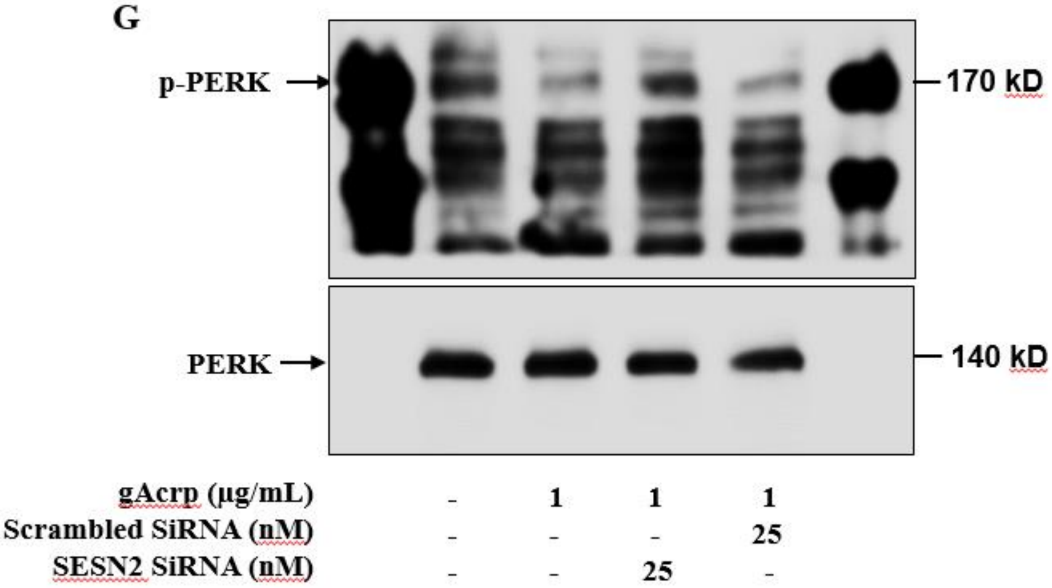

p-PERK
170 kD
PERK
140 kD
gAcrp (µg/mL) - 1 1 1
Scrambled SiRNA (nM) - - - 25
SESN2 SiRNA (nM) - - 25 -

H

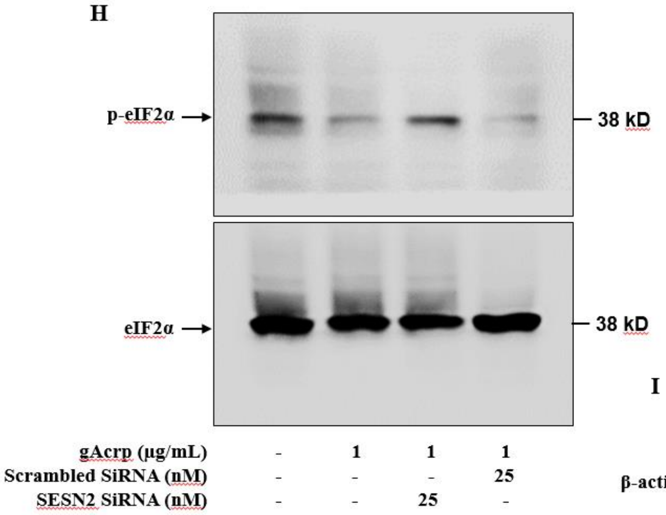

p-eIF2α
38 kD
eIF2α
38 kD
gAcrp (µg/mL) - 1 1 1
Scrambled SiRNA (nM) - - - 25
SESN2 SiRNA (nM) - - 25 -

I

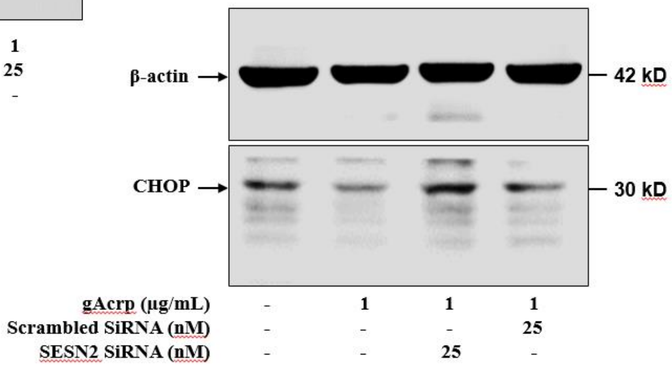

β-actin
42 kD
CHOP
30 kD
gAcrp (µg/mL) - 1 1 1
Scrambled SiRNA (nM) - - - 25
SESN2 SiRNA (nM) - - 25 -

Whole Western blots for Figure 5

F

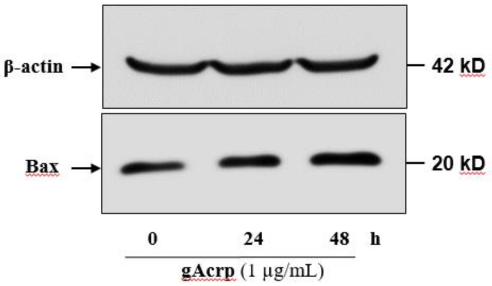

G

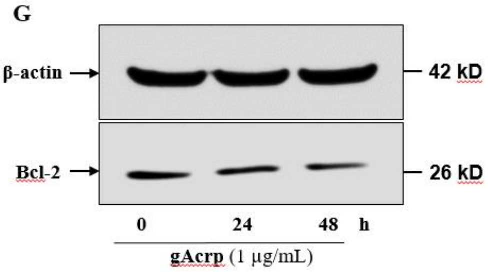

H

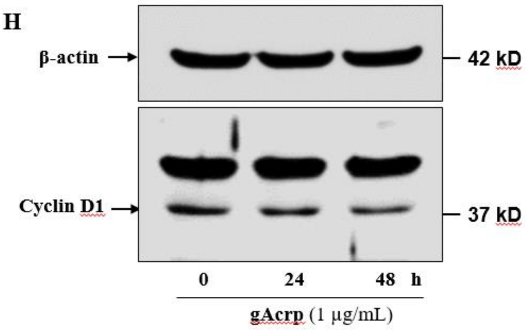

I/J

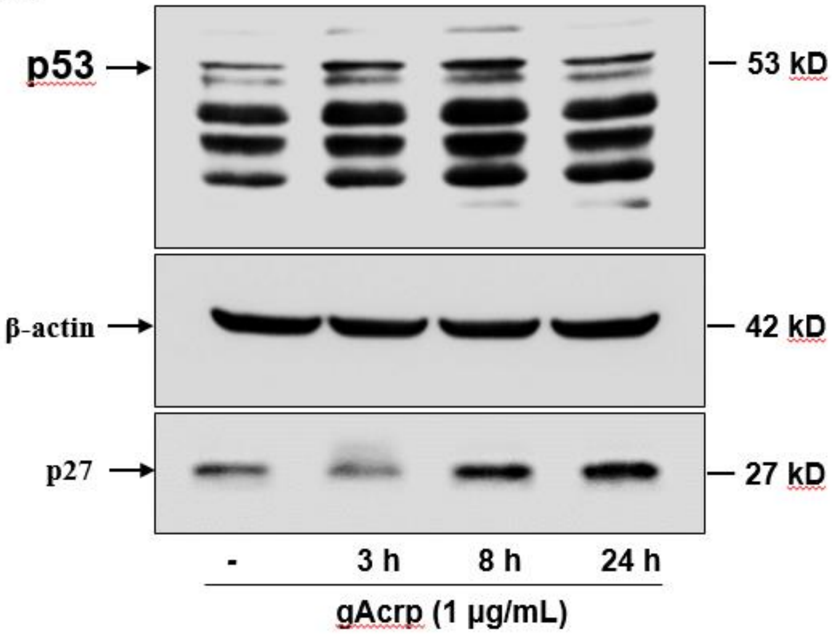

K

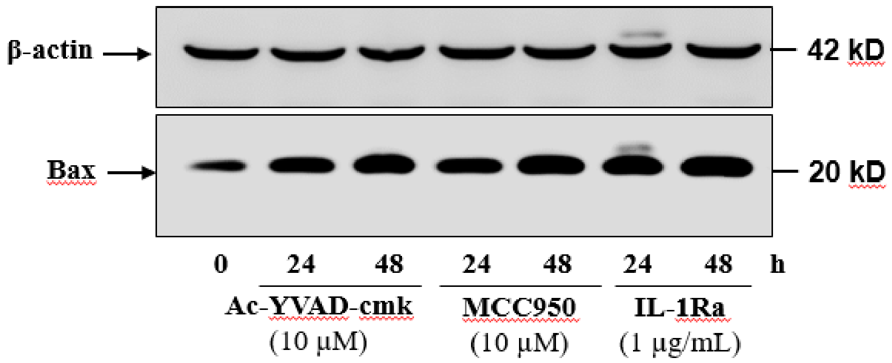
β-actin
Bax
42 kD
20 kD
0 24 48 24 48 24 48 h
Ac-YVAD-cmk
(10 µM)
MCC950
(10 µM)
IL-1Ra
(1 µg/mL)

L

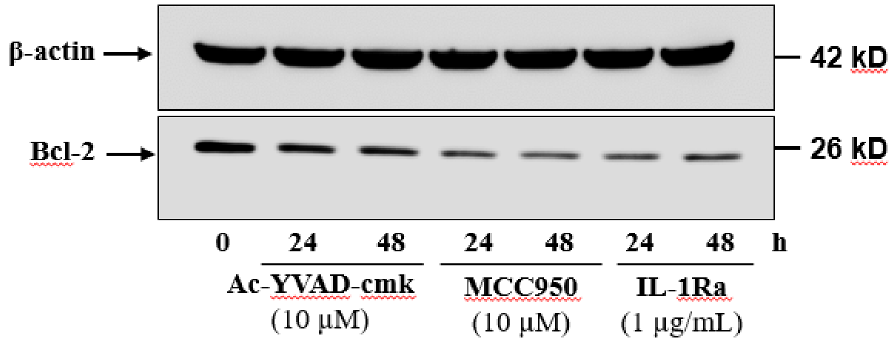
β-actin
Bcl-2
42 kD
26 kD
0 24 48 24 48 24 48 h
Ac-YVAD-cmk
(10 µM)
MCC950
(10 µM)
IL-1Ra
(1 µg/mL)

M

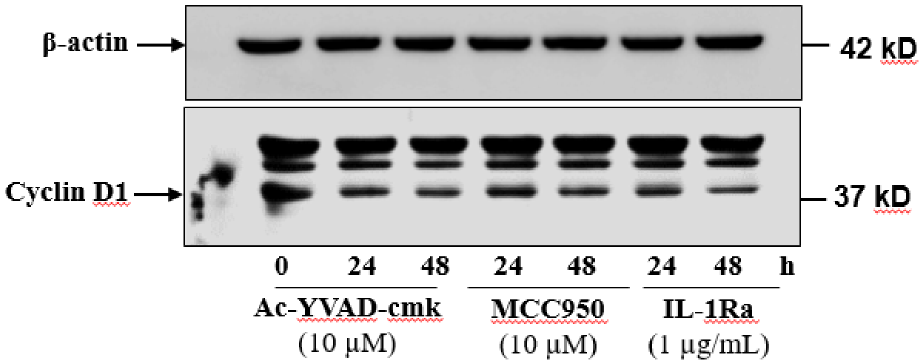
β-actin
Cyclin D1
42 kD
37 kD
0 24 48 24 48 24 48 h
Ac-YVAD-cmk
(10 µM)
MCC950
(10 µM)
IL-1Ra
(1 µg/mL)

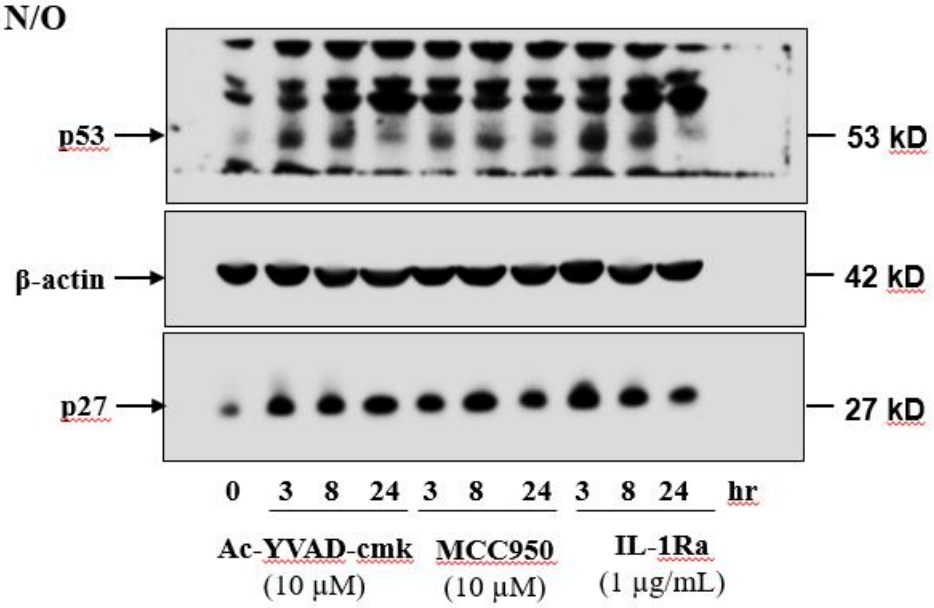

Whole Western blots for Figure 6

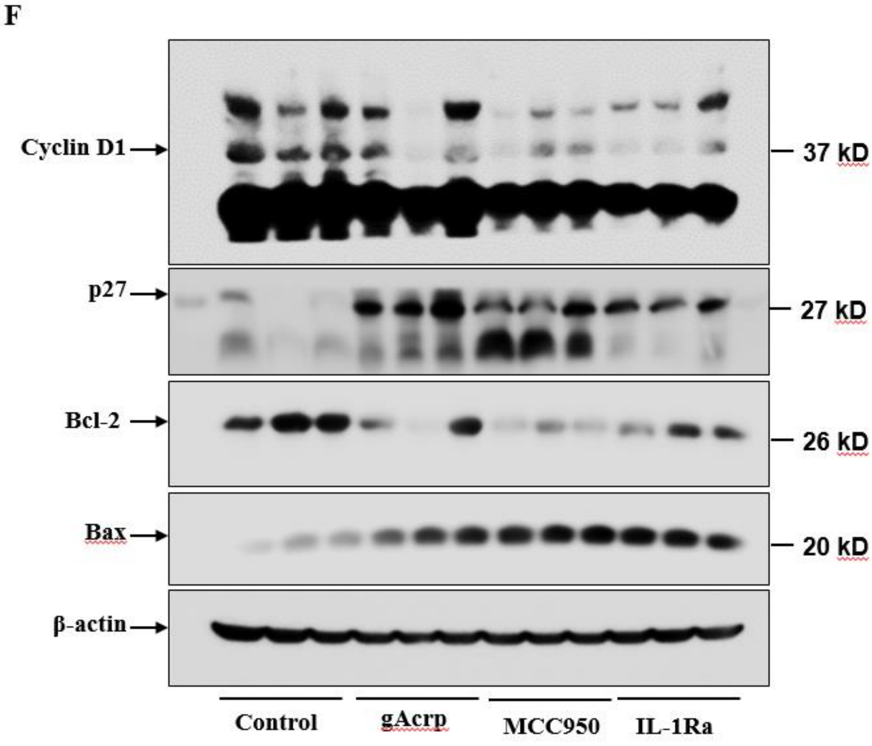

G

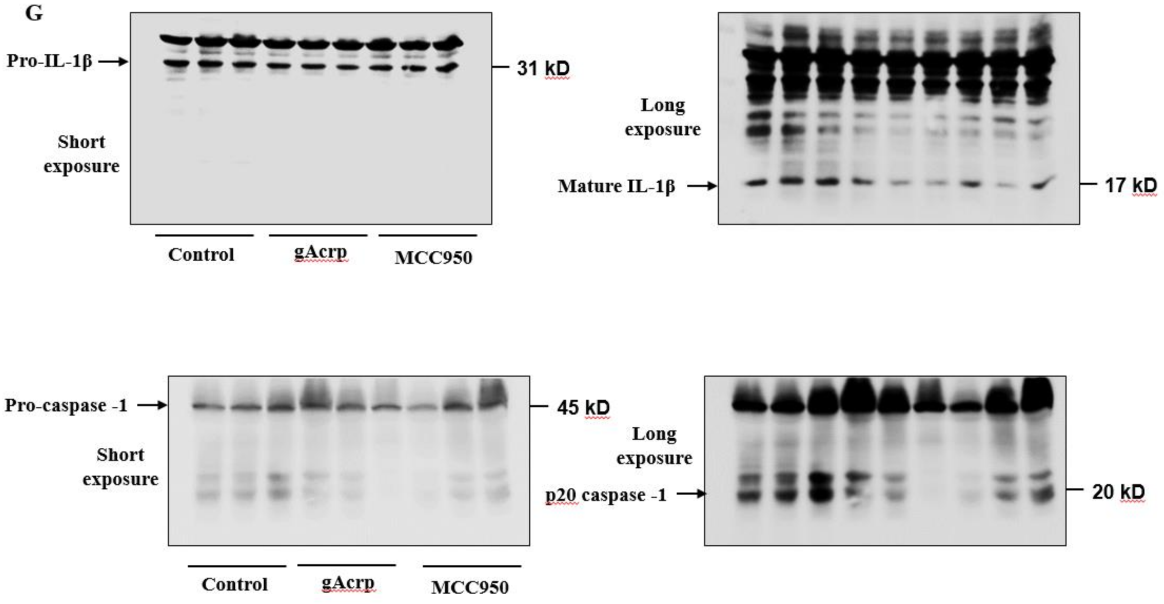

Pro-IL-1β
31 kD
Short exposure
Long exposure
Mature IL-1β
17 kD
Control
gAcrp
MCC950
Pro-caspase -1
45 kD
Short exposure
Long exposure
p20 caspase -1
20 kD
Control
gAcrp
MCC950

G/H

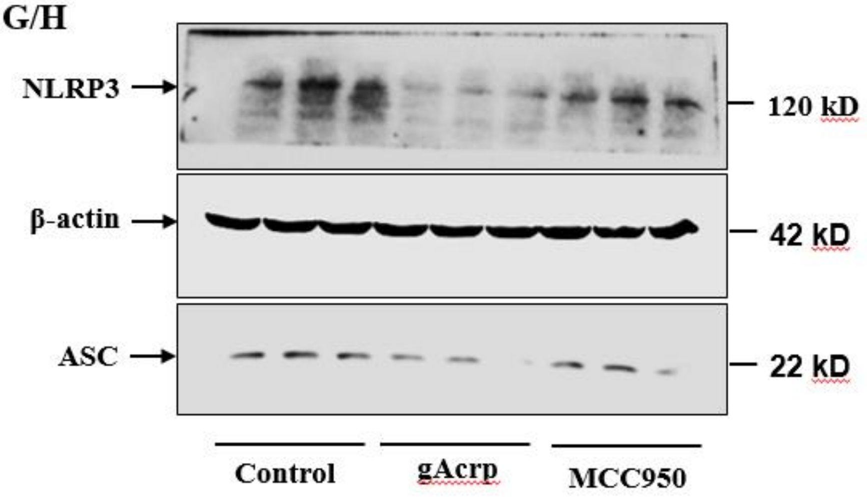

NLRP3
120 kD
β-actin
42 kD
ASC
22 kD
Control
gAcrp
MCC950

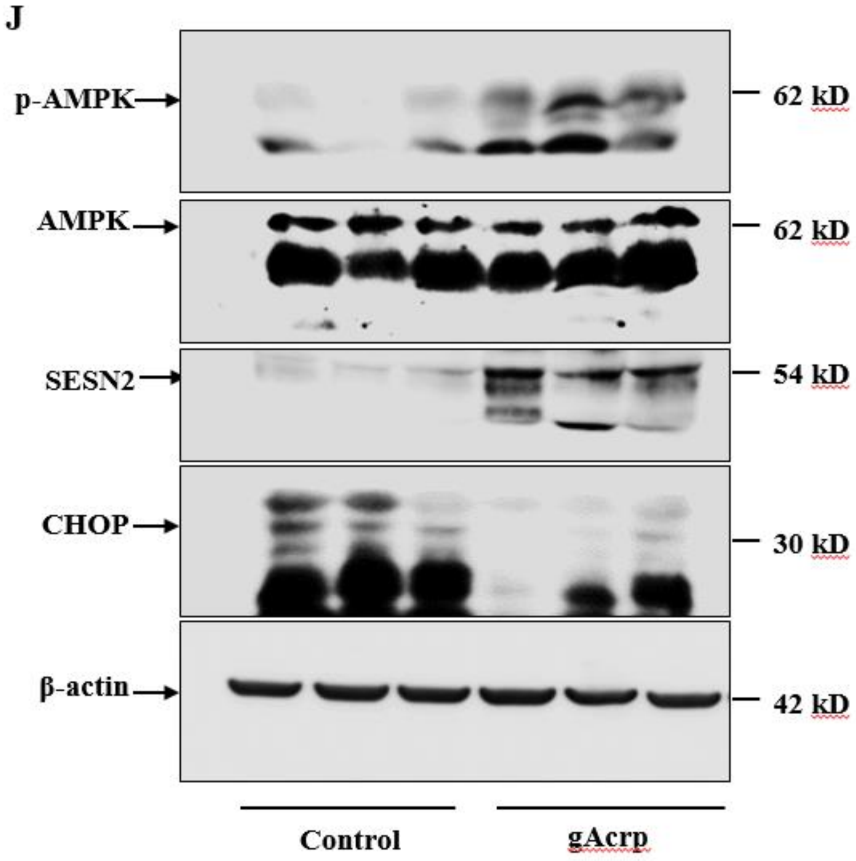

Figure S3. Blots from Western blot analysis.

© 2020 by the authors. Licensee MDPI, Basel, Switzerland. This article is an open access article distributed under the terms and conditions of the Creative Commons Attribution (CC BY) license (http://creativecommons.org/licenses/by/4.0/).
